# Unleashing the immune arsenal: development of broad spectrum multiepitope bluetongue vaccine targeting conserved T cell epitopes of structural proteins

**DOI:** 10.1186/s12864-025-12294-2

**Published:** 2026-04-17

**Authors:** Harish Babu Kolla, Anuj Kumar, Mansi Dutt, Roopa Hebbandi Nanjundappa, Karam Pal Singh, Peter Paul Clement Mertens, David Kelvin, Channakeshava Sokke Umeshappa

**Affiliations:** 1 Department of Microbiology and Immunology, Dalhousie University, Halifax, NS Canada; 2 IWK Health Center, Halifax, NS Canada; 3 Indian Veterinary Research Institute, Izatnagar, Uttar Pradesh India; 4 The University of Nottingham, Nottingham, UK; 5 Canada Research Chair in Human Immunology and Inflammation, Departments of Microbiology and Immunology, and Pediatrics Associate Member, Beatrice Hunter Cancer Research institute IWK Health Center, Dalhousie University, 5850-5980 University Ave, Halifax, NS B3K 6R8 Canada

**Keywords:** Bluetongue virus, Bluetongue serotypes, Structural proteins, Conserved epitopes, CD8 + T cell, CD4 + T cell, MHC, BoLA, *In Silico* Pan-BTV vaccine

## Abstract

Bluetongue (BT) is a severe arboviral disease affecting sheep, cows, and other wild ruminants, caused by the Bluetongue virus (BTV). The virus has evolved into over 32 serotypes, rendering existing vaccines less effective. While the structural proteins of this virus represent promising targets for vaccine development, they unfortunately exhibit high amino acid polymorphism and are laden with numerous inhibitory epitopes. However, certain structural proteins such as VP1 and VP7 are highly conserved and may contain epitopes capable of triggering cross-reactive cell-mediated immunity (CMI). In this study, we identified highly conserved MHC-I and -II-restricted T cell epitopes within VP1, VP5, and VP7 BTV proteins and designed multiepitope vaccine constructs using an in silico immunoinformatics pipeline for both laboratory mouse and bovine natural systems. The conserved epitopes utilized in the vaccines are highly antigenic, non-allergenic, non-toxic, and predicted to be capable of inducing IFN-𝛾. Both mouse and bovine vaccines were tethered with Toll-like receptor (TLR)-4-agonist adjuvants, beta-defensin 2–50 S ribosomal unit to stimulate innate immunity for the CMI development. Protein-protein docking analysis suggested favorable binding affinities between the vaccine constructs and TLR4, while 100-nanosecond molecular dynamics simulations supported the structural stability of the complexes. Although these computational findings are promising, all results require experimental validation. Future in vitro and in vivo studies are essential to confirm the immunogenicity, safety, and protective efficacy of the proposed vaccine candidates in target species.

**Supplementary Information:**

The online version contains supplementary material available at 10.1186/s12864-025-12294-2.

## Introduction

Bluetongue (BT) disease is widely distributed around the world and is one of the most economically important arboviral diseases of ruminants (primarily sheep). It is caused by bluetongue virus (BTV), belonging to genus *Orbivirus* within a new family *Sedoreoviridae* and the order *Reovirales* [1, 2]. BTV genome comprises 10 double-stranded RNA segments of various lengths (Seg-1 to Seg-10 in decreasing order of size), which encodes for the various proteins, such as Viral protein (VP) 1, VP2, VP3, VP4, VP5, VP6/6A, VP7 and Non-structural (NS) protein 1, NS2, and NS3/3A [3, 4]. Its infection induces severe damage to vascular endothelial cells, leading to edema, hemorrhage, vascular thrombosis, and tissue infarction [5, 6]. Animals affected by BTV typically experience lameness, weakness, reduced productivity, and varying degrees of mortality, resulting in considerable economic losses [6]. BTV can persist in the bloodstream for an extended period, raising concerns about the movement of animals across borders [6].

Vaccination is an effective measure to control the spread of BT infection among livestock [7–9]. Currently, there are more than 32 reported serotypes of BTV globally [https://www.woah.org/en/disease/bluetongue/, 10], including eight recently identified ‘novel’ types that may not infect vector insects. These newly emerged serotypes have the potential to exhibit unpredictable behavior, differing from the established 24 serotypes. The presence and widespread distribution of multiple BTV serotypes, along with the constant emergence, reemergence, and co-circulation of various serotypes, pose significant challenges to the effectiveness of existing commercially available BT vaccines including live attenuated vaccines (LAV) and inactivated vaccines (IAVs), which are predominantly serotype-specific [7–9, 11–13].

Despite significant advancements in BTV vaccine development, current approaches face critical limitations that hinder their widespread adoption and efficacy. Although the LAV BT vaccines are effective in providing protection for the sheep, the serious concerns with these vaccines, like reversion of virulence, high risk of abortions, and fetal malformations, limit their use [5, 8, 14–16]. On the other hand, the IAVs are relatively safer but confer weaker immune response and are also serotype-specific [5, 8, 9, 17–19]. DNA plasmid-vectored vaccines have shown efficacy in mouse models [20–24]; however, their practical deployment faces hurdles, including the risk of affecting genes responsible for cell growth, reduced immunogenicity, and high production costs for large-scale manufacturing [25]. Moreover, the recombinant vector-based vaccines, such as capripox, canarypox, adenovirus, and vaccinia virus, are developed by selectively targeting a particular antigen of interest [26–29]. These approaches could offer a limited protection against the other serotypes as only fewer conserved regions are present across the antigen. Another concern with these vaccines is regarding their safety as they are live attenuated viruses [30–32]. Being a potential immunogen, the vaccine candidates could be more toxic or exert other side effects. Virus-like particle (VLP) vaccines have shown some effectiveness [33].A study by Stwart et al. has shown that the BTV-8 VLPs are highly efficacious in controlling the viraemia induced by the BTV-8 isolate [34]. However, they have not been commercialized despite developed two to three decades ago. Moreover, they tend to induce serotype-specific humoral responses and require multiple doses. Hence, there is a pressing need to develop broad-spectrum commercially viable BT vaccines.

With the latest advances in computational resources, researchers have developed robust pipelines for designing vaccine candidates that are not only immunogenic and cross-reactive but also safe in terms of allergenicity and toxicity [35–37]. The multi-epitope-based vaccines developed from these resources are gaining importance in immunotherapy for infectious and cancers [38–44]. They are very efficient and feasible to express in a wide variety of expression systems such as *E. coli* [45], yeast [46], mammalian cells [47], and plants [48] using r-DNA technology [40, 49]. Previous studies on BT vaccine research highlight the importance of T-cell mediated cross reactive immune response against multiple BTV serotypes due to the presence of conserved T cell epitopes in the BTV antigens [50]. Despite these interesting observations, reports on the development of the multi-T cell epitope vaccines are lacking. Hence, to address the challenges associated with BT control and prevention and to harness T-cell-mediated cross-reactive response observed in BT, recently we developed innovative immunoinformatic-based pan-BTV vaccines in silico, utilizing conserved epitopes from non-structural proteins, NS1 and NS2 [51]. This vaccine design has potential to induce cross-protective T cell responses against all BTV serotypes. The nonstructural proteins are primarily involved in the virus’s replication and assembly processes and may not be as prominently recognized by the host immune system as the structural proteins. Furthermore, it is uncertain whether structural proteins harbor similar conserved epitopes for effective broad-spectrum vaccine development. Structural proteins, which form the viral capsid, play a crucial role in the virus’s ability to infect host cells and elicit strong immune responses. Therefore, the current study focuses on the structural proteins of BTV, specifically VP1, VP2, VP5, and VP7, which are highly conserved across multiple BTV serotypes [52, 53], making them strong candidates for the development of a pan-BTV vaccine. These structural proteins are well-documented immunogens and have been identified as effective vaccine targets in prior studies [54–57]. Furthermore, these proteins play critical roles in the BTV lifecycle, including viral entry, replication, and pathogenesis, underscoring their importance in eliciting an immune response [58, 59].

In this study, we focused on T cell epitopes rather than B cell epitopes due to the demonstrated ability of T cell epitopes to induce cross-protective, T cell-mediated immune responses across various viral serotypes, including BTV. This cross-reactivity is attributed to shared conserved determinants that underpin T cell-mediated immunity among BTV serotypes. In contrast, BTV infections predominantly elicit serotype-specific neutralizing antibodies targeting the outer capsid proteins, which are highly polymorphic across serotypes, thereby limiting their utility in developing a broad-spectrum vaccine.

**Fig. 1:**
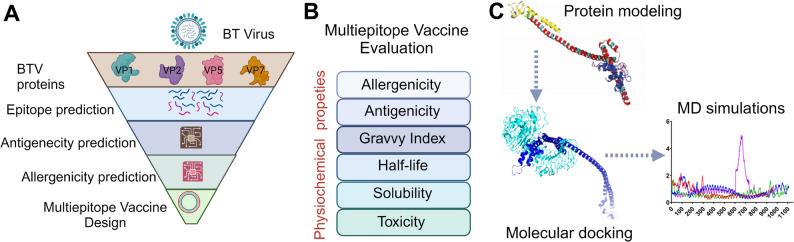
Schematic representation of the development process for a pan-BTV multivalent epitope vaccine against conventional BTV serotypes. **(A)** The process begins with selecting antigenic structural BTV proteins and predicting conserved T cell epitopes. These epitopes are then screened in silico for antigenicity, allergenicity, toxicity, and IFN-𝛾-inducing abilities. **(B)** Next, a multi-epitope pan-BTV vaccine is designed. The vaccine undergoes rigorous in silico screening tests to evaluate its safety and antigenicity. **(C)** Various vaccine constructs are comprehensively assessed for stability, which includes 3D structure modeling, molecular docking, and simulations with bovine TLR4 to determine affinity and interaction with the host

Here, we aim to investigate the feasibility of developing a pan-BTV vaccine by targeting the structural proteins of BTV. We employed in silico methods, such as multiple sequence alignments and epitope mapping, to analyze the key structural proteins—VP1, VP2, VP5, and VP7—of all the BTV serotypes, sourced from the National Center for Biotechnology Information (NCBI) database. We specifically analyzed these proteins for the presence of conserved T cell epitopes recognized by murine Major Histocompatibility Complex (MHC) and Bovine Leukocyte Antigen (BoLA) molecules and identified many conserved CD8 + and CD4 + T cell epitopes in VP1 and VP7 proteins **(**Fig. 1**)**. These epitopes along with TLR4 agonists were utilized in the development of in silico-based pan-BTV vaccines. Protein-protein docking analysis demonstrated robust binding affinities and extensive 100-nanosecond molecular dynamics simulations confirmed the stability of the complexes formed between the vaccine structures and TLR4. This comprehensive study serves as a proof of concept for future research efforts focused on creating potent pan-BTV vaccines **(**Fig. 1**)**, which have the potential to offer cross-protection against all known BTV serotypes, thereby mitigating the dissemination of these virulent strains and aiding in the control and prevention of BT.

## Methodology

### Sequence retrieval and multiple sequence alignment

We performed multiple sequence alignment to determine the amino acid sequence homology in the structural proteins of BTV in order to identify the conserved T cell epitopes based on the amino acid identity. We mined the full length amino acid sequences of the BTV structural proteins, such as VP1, VP2, VP5 and VP7, from all the 24 serotypes out of 32 that are only available in NCBI taxonomy browser (https://www.ncbi.nlm.nih.gov/Taxonomy/Browser/wwwtax.cgi.id=40051*).* Later, we aligned the amino acid sequences of all the 4 proteins using BLOSUM 65 matrix of ClustalW module in the Molecular evolutionary genetic analysis (MEGA-X) software. The alignment files of all the proteins were saved in MEGA format for further analysis. Then the amino acid sequence of all the proteins was determined using the MEGA files.

### Prediction of murine and bovine MHC I- and II-restricted epitopes

Later we predicted the T cell epitopes in the VP1, VP2, VP5 and VP7 proteins for mouse and bovine systems. We selected the mouse model for its increasing utility in studying Bluetongue (BT), including assessing vaccine efficacy, while bovines were chosen due to their status as natural hosts susceptible to BTV infection, akin to sheep. The MHC class I and II specific CD8 + and CD4 + T cell epitopes were identified in VP1, VP2, VP5 and VP7 proteins **(**Fig. 1A**)**. Later, the conserved T cells were identified based on their amino acid sequence conservation among all 24 BTV serotypes. For epitope prediction, we used the amino acid sequences of these 4 proteins from BTV-1 serotype. For the prediction of CD8 + T cell epitopes, we used “NetMHCpan BA 4.1” module of Immune Epitope Database Epitope Analysis Resource (IEDB-AR) website (http://tools.iedb.org/mhci/) [60]. The epitopes were predicted corresponding to the MHC class I alleles (H2-Kb and H2-Db) in C57BL/6 mice. The host was selected as mouse for the epitope prediction and 9-mer CD8 + epitopes were used in our analysis as they are the most commonly presented by MHC class I molecules influenced by three key antigen processing mechanisms such as proteasomal cleavage of precursor proteins, peptide transport into the endoplasmic reticulum via the TAP transporter, and further peptide trimming by ERAP [61]. The epitopes were selected based on their half maximal inhibitory concentration (IC50) values, which is an indirect measure of how strongly the peptide binds to the MHC molecule. Epitope selection relied on with a threshold of < 500nM set for CD8 + T cell epitopes [62]. Likewise, predictions for CD4 + T cell epitopes were conducted for the MHC II H2-IA^b^ allele in C57BL/6 mice, employing the “MHCIIpan 4.0 BA” module accessible on the IEDB-AR tool website (http://tools.iedb.org/mhcii/) [60]. Threshold parameters for CD4 + T cell epitopes were set using IC50 values, with < 1000nM being considered, which indicates the strong interaction between the CD4 + T cell epitope peptide and the MHC allele [63, 64].

Likewise, we predicted the CD8 + and CD4 + T cell epitopes corresponding to the bovine immune system **(**Fig. 1A**)**. The prediction was carried out utilizing the IEDB-AR server (http://tools.iedb.org/mhci/) [65], with the host species specified as “cow.” While predicting the CD8 + T cell epitopes, the most frequent BoLA class I alleles such as BoLA-BoLA-1*02301, BoLA-2*01201, BoLA-3*00201, BoLA-4*02401, BoLA-6*01301 and BoLA-6*01302 were selected [66, 67]. For predicting CD4 + T cell epitopes, we used NetBOLAIIPan 1.0 (https://services.healthtech.dtu.dk/service.php? NetBoLAIIpan-1.0) [68]. A threshold IC50 value of < 500nM was utilized for epitope prediction as mentioned above for the mouse CD8 + T cell epitope prediction. The CD4 + T cell epitopes were predicted corresponding all the BoLA II alleles that were deposited in the tool (BoLA-BoLA-DRB3_0101, BoLA-DRB3_1001, BoLADRB3_1101, BoLA-DRB3_1201, BoLA-DRB3_1501, BoLADRB3_1601 and BoLADRB3_2002). The tool generates potential predicted T cell epitopes in 15 amino acid length, each with different % Rank EL scores. A threshold parameter of % Rank EL scores less than 1.0 was utilized for the prediction of CD4 + T cell epitopes [68].

### Analysis of the epitopes for pan-BTV vaccine development

To obtain conserved CD8 + and CD4 + T cell epitopes, we examined the predicted epitopes within the VP1, VP2, VP5 and VP7 proteins of BTV1 based on their amino acid sequence identity by comparing them with amino acid alignment files previously generated using MEGA-X software. Our analysis aimed to determine the extent of conservation of these epitopes within the alignment files encompassing VP1, VP2, VP5 and VP7 proteins across all 24 serotypes. The conserved T cell epitopes were further screened before designing a multi-epitope vaccine **(**Fig. 1A**)**. These epitopes were subjected for screening based on their antigenicity, allergenticity, toxicity and their predicted ability to induce IFN-𝛾 in order to identify potential epitopes which are antigenic in nature and doesn’t exert their allergic or toxic nature. Firstly, the antigenicity of these epitopes was predicted using VaxiJen v2.0 server (http://www.ddg-pharmfac.net/vaxijen/VaxiJen/VaxiJen.html) with a default threshold of 0.4 [69]. The epitopes with the prediction score of above 0.4 are considered as antigens. Similarly, allergenicity and toxicity of these epitopes was predicted using online resources called AllerTOP v2.0 (https://www.ddg-pharmfac.net/AllerTOP/) [70, 71] and ToxinPred (https://webs.iiitd.edu.in/raghava/toxinpred/algo.php) [72] with the default threshold or settings. Finally, the IFN-𝛾-inducing potential of the epitopes was determined with the help of design module in the IFNepitope tool (https://webs.iiitd.edu.in/raghava/ifnepitope/application.php) [73].

### Designing pan-BTV vaccine

Following a rigorous epitope screening process, those conserved epitopes that were predicted to exhibit antigenicity, non-allergenicity, non-toxicity, and IFN-𝛾-inducing properties were selected for the development of a pan-BTV multi-epitope vaccine capable of eliciting an immune response across all serotypes **(**Fig. 1B**)**. During vaccine design, the validated linkers [74] were utilized to connect the CD8 + T cell epitopes using the ‘AAY’ linker as it allows the C terminal cleavage of the peptide and does not inhibit N terminal cleavage of the downstream epitope. Moreover, the C terminal Y residue in the linker allows binding to TAP transporter to allow presentation of the peptide [75, 76]. The CD4 + T cell epitopes were joined with the ‘GPGPG’ linker as it doesn’t bind effectively to the MHC II molecules and prevents the junctional immunogenicity against the CD4 + T cell epitopes during antigen processing [77]. To enhance the effectiveness of the designed vaccines, an adjuvant sequence was incorporated at the N-terminal end of the vaccine using the ‘EAAAK’ linker. This linker provides flexibility and appropriate spacing to ensure effective antigen processing and structural integrity, as supported by previous studies [51, 78–81]. We employed two TLR4-activating adjuvants, beta-defensin 2 and a 50 S ribosome subunit, as carriers in our vaccine constructs to promote dendritic cell maturation and initiate a robust cell-mediated immune response in vivo [82–90].

### Antigenicity, allergenicity, solubility, Immunological, and physicochemical properties of the vaccine constructs

We devised a total of 4 vaccine constructs, comprising mouse and bovine vaccines, each incorporating two distinct TLR4 agonist adjuvants. These vaccines underwent evaluation for antigenicity and allergenicity as previously described. Immune simulations were performed to determine the ability of vaccine constructs to induce immune response under the simulated environment as described in our previous study [51]. Immune simulations were conducted using the C-IMMSIM server (https://kraken.iac.rm.cnr.it/C-IMMSIM/index.php? page=1), which currently supports only the human immune system. Due to the lack of immune simulation tools tailored for BT’s natural hosts, such as bovine or ovine models, we performed the analysis using the human system as a proxy. The simulation was run with default parameters, with minor modifications: the total number of simulation steps was set to 1050, and vaccine injections were administered at steps 1, 84, and 168, as previously described in the literature [51]. Additionally, the solubility and physicochemical properties, such as molecular weight, theoretical isoelectric point (pI), instability index (II), aliphatic index (AI), and grand average of hydropathicity index (GRAVY), were predicted using the Protein-Sol (http://protein-sol.manchester.ac.uk/) [91] and ProtParam tool (physico-chemical properties) (https://web.expasy.org/protparam/*)* [92], respectively. The vaccine constructs were further assessed for their ability to be expressed in heterologous host such as *E. coli* for recombineering in terms of Codon adaptation index and % GC content as we followed in the previous study [51].

### Structure modeling and evaluation

We employed an in-silico protein modeling approach to predict the three-dimensional structures of the four vaccines for the molecular docking studies and structural analysis **(**Fig. 1C**)**. The 3-D structures of the vaccine constructs were modeled using the Robetta server (https://robetta.bakerlab.org/) [93]. Subsequently, these protein models underwent refinement using the GalaxyWEB server (https://galaxy.seoklab.org/) [94] and the quality of the models was assessed based on the phi (ϕ) and psi (Ψ) torsion angles using the PROCHECK module ([95] within the protein structure verification server (PSVS) (https://montelionelab.chem.rpi.edu/PSVS/PSVS/*).*

### Molecular Docking

Molecular docking studies were conducted to assess the interaction between the structures of vaccine proteins and TLR4, a potent inducer of antiviral activity [96], as described previously **(**Fig. 1C**)**. Briefly, the X-ray crystallography-derived structures of mouse TLR4, identified by the PDB ID 4G8A ID, were obtained from the RCSB-PDB (https://www.rcsb.org/). As the crystal structures of bovine TLR4 are unavailable in the PDB, we retrieved modeled structures from the alpha-fold database for bovine TLR4 (Q9GL65). The receptor molecule structure was prepared for molecular docking by eliminating heteroatoms and water molecules using PyMOL (https://www.pymol.org/). Molecular docking studies were performed using the ClusPro 2.0 server (https://cluspro.bu.edu/publications.php) [97] with chain ‘A’ assigned to receptors (TLR4) and chain ‘B’ to the vaccine constructs as per the requirement of the server. The best docking poses were selected based on the top-ranking docking scores, and the largest cluster size. Further, the binding affinity or Gibbs free energy between TLR4 and vaccine constructs in each docking complex was assessed using the PRODIGY (PROtein binDIng enerGY prediction) server (https://wenmr.science.uu.nl/prodigy/) [98], while two-dimensional interactions were visualized using the PDBsum tool (https://www.ebi.ac.uk/thornton-srv/databases/pdbsum/) [99].

### Molecular dynamics simulations

To assess the stability of the docked complexes at the atomic level, the molecular dynamics (MD) simulations were carried out for all four complexes. The AMBER99SB-ILDN protein force field [100] integrated into the GROMACS 2023.1 package [101], utilized to perform MDS on high-performance computing (HPC) facility. The complexes were immersed in solvent using the transferable intermolecular potential 3P (TIP3P) water model and underwent energy minimization with LINear Constraint Solver (LINCS) constraint algorithms to achieve neutrality [102]. Subsequent equilibration steps involved NVT ensemble at 300 K and NPT ensemble with the Parinello-Rahman barostat coupling ensembles [103]. Following equilibration, a 100ns MDS production run was performed for all four docked complexes with a time step of 2 fsas previously described in multi-epitope vaccine development studies [104–108]. After MDS, trajectories were analyzed, and plots were generated using a set of modules embedded in the GROMACS package.

## Results

### Predicted frequencies of T cell epitopes in the structural proteins of BTV-1

Multiple sequence alignment analysis revealed that the VP1 and VP7 displayed high conservation levels, with sequence similarities of 95.6% and 73.63%, respectively, indicating the presence of conserved epitopes within these proteins. In contrast, VP5 exhibited moderate variability, with an identity of 39.69%, making it the second most variable protein, while VP2 showed the lowest identity at 10.25%, consistent with previous research [109, 110].

**Fig. 2:**
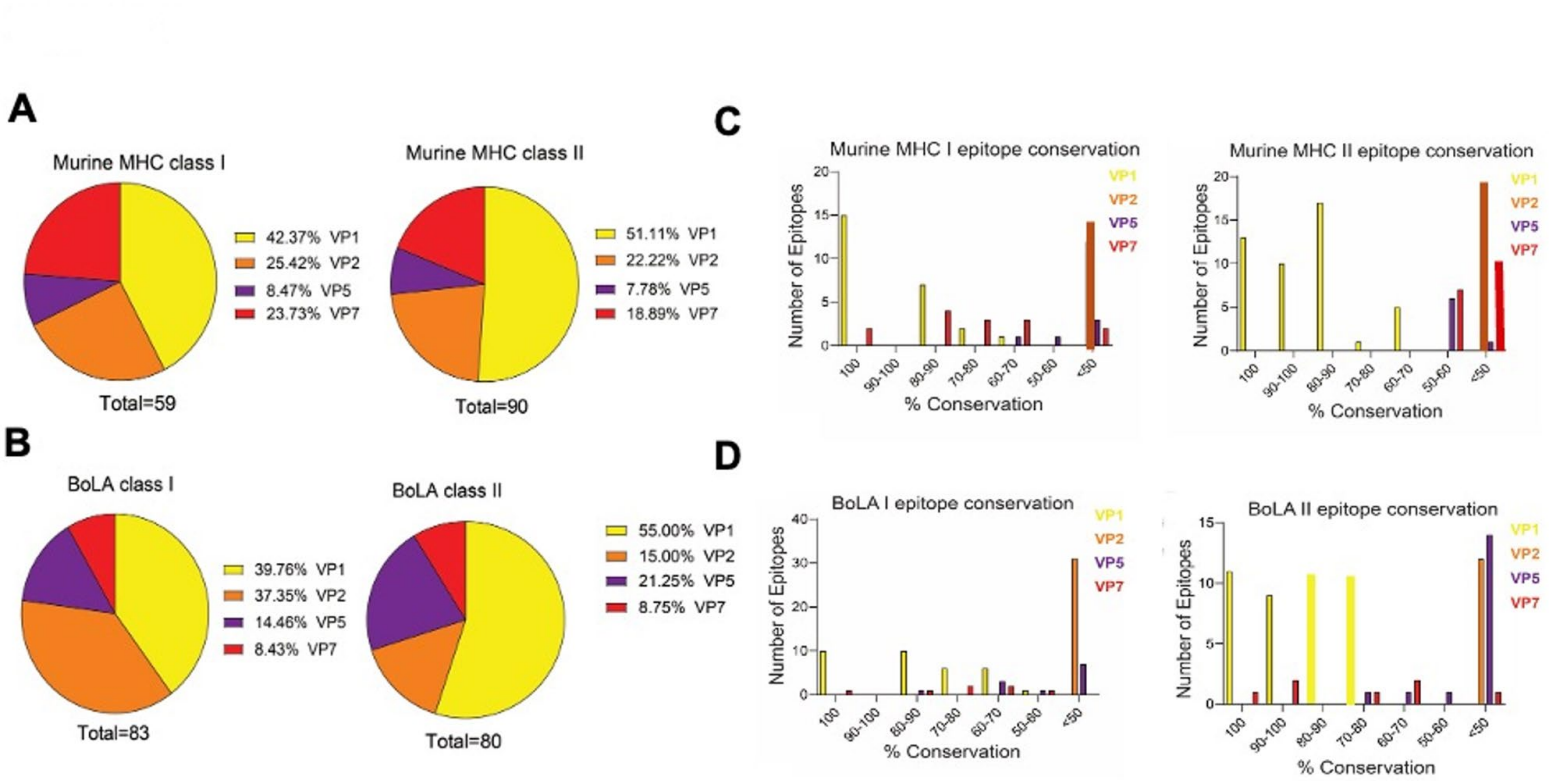
Prediction of the conserved murine- and bovine-specific T cell epitopes among the structural proteins of BTV serotypes. A-B. Distribution of murine MHC. (**A**) and bovine BoLA. (**B**)-restricted CD8 + and CD4 + T cell epitopes in the structural proteins of BTV. (**C-D**) Bar graph presenting the degree of amino acid conservation (%) among murine MHC class I- and II-restricted. (**C**) and bovine BoLA class I- and II-restricted. (**D**) CD8 + and CD4 + T cell epitopes in BTV structural proteins

Later we, predicted the CD8 + T cell epitopes recognized by H-2-D^b^ and H-2-K^b^ class I molecules, as well as CD4 + T cell epitopes recognized by H-2-IA^b^ class II molecule T cell epitopes in the structural proteins of BTV-1 as these alleles are from the C57BL/6 mouse background and it has been widely utilized to investigate the immunopathogenesis of BTV and assess vaccine candidates against the virus. Our predictions yielded a total of 59 CD8 + T cell epitopes for the mouse system, distributed as follows: 42.37% in VP1 (total 25 @ 0.17 amino acids per kDa, data not shown in the figure), 25.42% in VP2 (total 15 @ 0.17 per kDa, data not shown in the figure), 5 (8.47%) in VP5 @ 0.13 per kDa (data not shown in the figure), and 14 (23.73%) in VP7 proteins of BTV-1 @ 0.37 per kDa (data not shown in the figure) **(**Fig. 2A, Table S1**)**. On the other hand, we identified 90 CD4 + T cell epitopes, with VP1 harboring the majority, accounting for 46 (51.11%) of the epitopes @ 0.30 per kDa (data not shown in the figure), followed by VP2 with 20 (22.22%) @ 0.23 per kDa (data not shown in the figure), VP5 with 7 (7.78%) @ 0.18 per kDa (data not shown in the figure), and VP7 with 17 (18.89%) epitopes @ 0.45 per kDa (data not shown in the figure) **(**Fig. 2A, Table S2**).** These findings highlight VP1 as the primary source of both mouse CD8 + and CD4 + T cell epitopes.

For developing bovine-specific BT vaccines, we considered the most frequent BoLA class I alleles, including BoLA-6*01301*,* BoLA-6*01302, BoLA-2*01201*,* BoLA-4*02401, BoLA-3*00101*,* and BoLA-1*02301, for CD8 + T cell epitope prediction. We identified a total of 83 CD8 + T cell epitopes, with 33 (39.75%) in VP1 @ 0.22 per kDa (data not shown in the figure), 31 (37.34%) in VP2 @ 0.36 per kDa (data nost shown in the figure), 12 (14.45%) in VP5 @ 0.30 per kDa (data not shown in the figure), and 7 (8.43%) in VP7 proteins of BTV1 @ 0.18 per kDa (data not shown in the figure) **(**Fig. 2B, Table S3). For CD4 + T cell epitopes, we found 44 (55%) in VP1 @ 0.30 per kDa (data not shown in the figure), 12 (15%) in VP2 at 0.14 per kDa (data not shown in the figure), 17 (21.25%) in VP5 @ 0.43 per kDa (data not shown in the figure), and 7 (8.75%) in VP7 of the BTV1 serotype @ 0.19 per kDa (data not shown in the figure) (Fig. 2B, Table S4). Strikingly, these findings suggest that VP1 is the primary source of bovine CD8 + and CD4 + T cell epitopes, like the mouse system.

### Predicted conserved T cell epitopes among the BTV serotypes

All the predicted CD8 + and CD4 + T cell epitopes in VP1 exhibited substantial amino acid identity throughout the peptide sequence, with a minimum of 66.66% amino acid sequence. Remarkably, VP1 contained 15 CD8 + T cell epitopes that were 100% conserved, with the remaining epitopes displaying conservation levels ranging from 66.66% to 88.88% **(**Fig. 2C**)**. Similarly, VP1 harbored 13 CD4 + T cell epitopes that were 100% conserved, along with others exhibiting conservation levels ranging from 66.66% to 93.33% **(**Fig. 2C**)**. In contrast, the VP1 protein exhibited 13 CD4 + T cell epitopes with 100% amino acid conservation, followed by 9 epitopes with 93.33%, 18 with 86.66%, 1 with 73.33%, and 5 with 66.66% conservation **(**Fig. 2C**)**. Regarding the VP7 protein, 2 CD8 + T cell epitopes were found to be 100% conserved, with 4 epitopes exhibiting 88.88% conservation, 3 with 77.77%, and 3 with 66.66% (Fig. 2C**)**. Additionally, two CD8 + T cell epitopes displayed conservation levels below 50%, with conservation rates of 33.33% and 22.22% (Fig. 2C). Consistent with expectations, no conserved CD8 + and CD4 + T cell epitopes were detected in the VP2 protein **(**Fig. 2C). Despite amino acid variability in the VP5 protein, one CD8 + T cell epitope with 66.66% conservation **(**Fig. 2C) and 2 CD4 + T cell epitopes with 60% conservation were identified in this protein (Fig. 2C).

Similar to the CD8 + and CD4 + T cell epitopes recognized by the murine system; we observed a comparable pattern of conserved epitopes in the bovine system (Fig. 2D; Table S3). The VP1 protein exhibited 10 BoLA class I-specific CD8 + T cell epitopes that were 100% conserved, with an additional 10 epitopes displaying 88.88% conservation, while 7 epitopes exhibited 77.77% conservation and another 6 had 66.66% conservation. A similar trend of CD8 + T cell epitope conservation was observed in the VP7 protein, which included 1 epitope each with 100%, 88.88%, and 55.55%, and 2 epitopes each with 77.77% and 66.66% (Fig. 2D). The VP7 protein also contained 1 CD4 + T cell epitope that was 100% conserved, along with 2 epitopes with 93.33%, 1 with 73.33%, and 2 with 66.66% conservation, covering all 24 BTV serotypes (Fig. 2D). As expected, no conserved CD8 + and CD4 + T cell epitopes were found in VP2 (Fig. 2D). Surprisingly, we identified 4 CD8 + T cell epitopes, 1 with 88.88% and 3 epitopes with 66.66% conservation in the VP5 protein (Fig. 2D the second most variable structural protein after VP2. In contrast, there were few CD4 + T cell epitopes in the protein, which were highly conserved to a certain extent (1 with 73.33%, 1 with 66.66%, and 1 with 60%) (Fig. 2D; Table S4).

### Selection of predicted conserved epitopes and design of pan-BTV vaccine

**Fig. 3:**
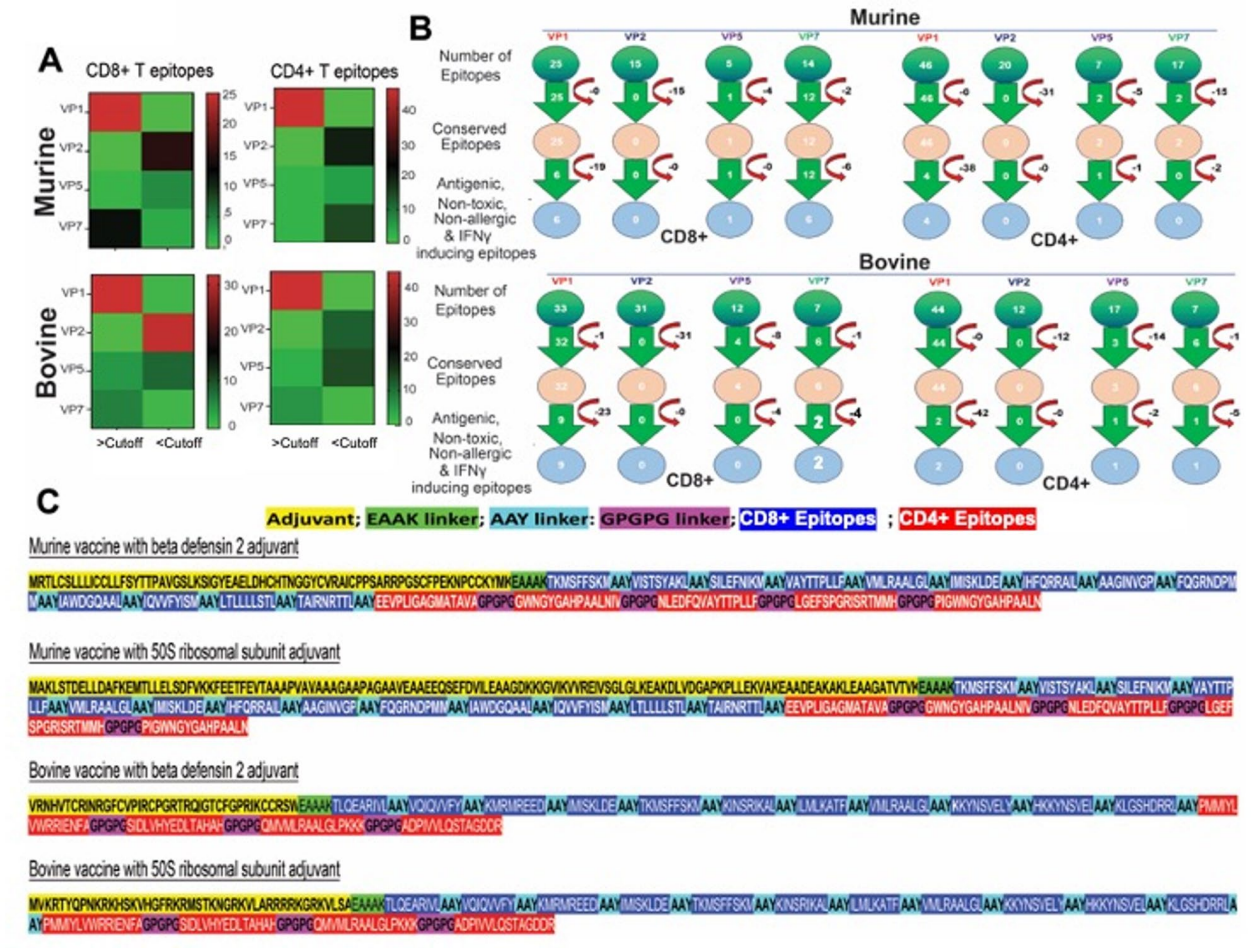
Conserved epitopes among the structural proteins of BTV and the design of a broad-spectrum BTV multi-epitope vaccine. **A** The VP1 and VP7 are the hotspots for conserved epitopes in both murine and bovine systems. The heat maps show a predominance of CD4 + and CD8 + T cell epitopes, particularly within the VP1 protein. **B** In silico screening of T cell epitopes for the design of a broad-spectrum BTV vaccine. **C** Molecular design of a broad-spectrum BTV multi-epitope vaccine corresponding to murine and bovine systems. The CD8 + and CD4 + T cell epitopes were joined together with AAY and GPGPG linkers, respectively, whereas adjuvants are joined at the N-terminal of the vaccine construct using EAAAK linker

From the above analysis, we observed a higher frequency of conserved CD8 + and CD4 + T cell epitopes in both murine and bovine systems within the VP1 followed by VP7 proteins **(**Fig. 3A**)**, indicating that these proteins are potential targets for consideration in BT vaccine development. The screening process yielded a total of 13 mouse CD8 + T cell epitopes—6 from VP1, 1 from VP5, and 6 from VP7 proteins—that met the criteria of being antigenic, non-allergenic, non-toxic, and predicted to be IFN-𝛾 inducers **(**Fig. 3B**)**. Similarly, we identified 5 CD4 + T cell epitopes—4 in VP1 and 1 in VP5 protein—that satisfied these criteria (Fig. 3B). For the design of the bovine vaccine, we obtained a total of 11 CD8 + T cell epitopes—9 from VP1 and 2 from VP7 proteins **(**Fig. 3B**)** —as well as 4 CD4 + T cell epitopes, 2 from VP1, and 1 each from VP5 and VP7 proteins (Fig. 3B).

Although a CD4 + T cell epitope from VP5 protein exhibits only moderate sequence conservation of ~ 40%, it was included in the vaccine design due to its distribution across a broad range of serotypes. Specifically, this epitope is conserved at or above 40% in all serotypes, making it one of the few epitopes with partial representation across diverse viral strains. Including this epitope enhances the overall breadth of CD4 + T cell coverage, contributing to a more diverse and potentially cross-protective helper T cell response. This strategy is particularly important for multivalent vaccine design in species such as cattle, where broad serotype representation is necessary due to natural exposure to multiple serotypes in the field. Moreover, while absolute sequence conservation is limited, CD4 + T cell epitopes can tolerate certain degrees of variation, especially when critical MHC-binding residues and TCR contact points are preserved. The inclusion of this VP5 epitope, therefore, strategically boosts the number of helper T cell targets while maintaining relevance across circulating strains.

The selected epitopes, which demonstrated predicted potent anti-viral T cell responses, were utilized to design in silico multi-epitope pan-BTV vaccines, as described in the Methods section (Fig. 3C). Briefly, the conserved T cell epitopes were connected using suitable linkers, and TLR4 agonist adjuvants, such as beta-defensin 2–50 s ribosomal protein, were integrated into the N-terminus region of the CD8 + CTL and CD4 + T helper polyepitopic region to boost immunogenicity [111]. With this approach, we developed 2 constructs for mice and 2 for bovines, each utilizing a distinct TLR4 agonist adjuvant: mouse vaccines with either beta-defensin 2 (mVac-𝛽-def) or 50 S ribosomal subunit (mVac-50SR) adjuvants, and bovine vaccines with either beta-defensin 2 (bVac-𝛽-def) or 50 S ribosomal protein (bVac-50SR) adjuvants.

### Evaluation of vaccine constructs

The physicochemical properties of the vaccine constructs were analyzed, including molecular weights, theoretical pI, instability index, and GRAVY scores. The mVac-𝛽-def, mVac-50SR, bVac-𝛽-def, and bVac-50SR constructs have molecular weights of 34738.56, 40358.78, 28107.17, and 28892.16, respectively, with theoretical pI values of 8.86, 5.41, 9.79, and 10.25 **(**Table 1**)**. Notably, all constructs exhibit stability, with instability index values of 33.67, 27.77, 36.24, and 44.65, respectively, although bVac-50SR appears slightly less stable compared to the others **(**Table 1**)**. Additionally, GRAVY scores (measures the sum of hydropathy values of all amino acids in the protein) for all four constructs are 0.363, 0.353, 0.042, and − 0.183, indicating the mouse vaccines are hydrophobic while bovine vaccines, bVac-𝛽-def, and bVac-50SR, are neutral and hydrophilic in nature **(**Table 1**)**. Antigenicity profiling reveals scores of 0.6385, 0.5678, 0.5736, and 0.5501, respectively, indicating high antigenicity for all vaccines **(**Table 1**)**. Lastly, allergenicity profiling confirms that all vaccine constructs are non-allergenic **(**Table 1**)**.

**Table 1:** Immunological and physicochemical attributes of all the designed vaccine constructs

Vaccine	Description	Antigenicity Score	Allergenicity	Molecular weight (Daltons)	Isoelectric point	Instability index	GRAVY
mVac-β-def	Mouse vaccine with beta defensin adjuvant	0.6385	Non-allergen	34738.56	8.86	33.67	0.363
mVac-50SR	Mouse vaccine with 50 S ribosomal adjuvant	0.5678	Non-allergen	40358.78	5.41	27.77	0.353
bVac-β-def	Bovine vaccine with beta defensin adjuvant	0.5736	Non-allergen	28107.17	9.79	36.24	0.042
bVac-50SR	Bovine vaccine with 50 S ribosomal adjuvant	0.5501	Non-allergen	28892.16	10.25	44.65	−0.183

**Fig. 4:**
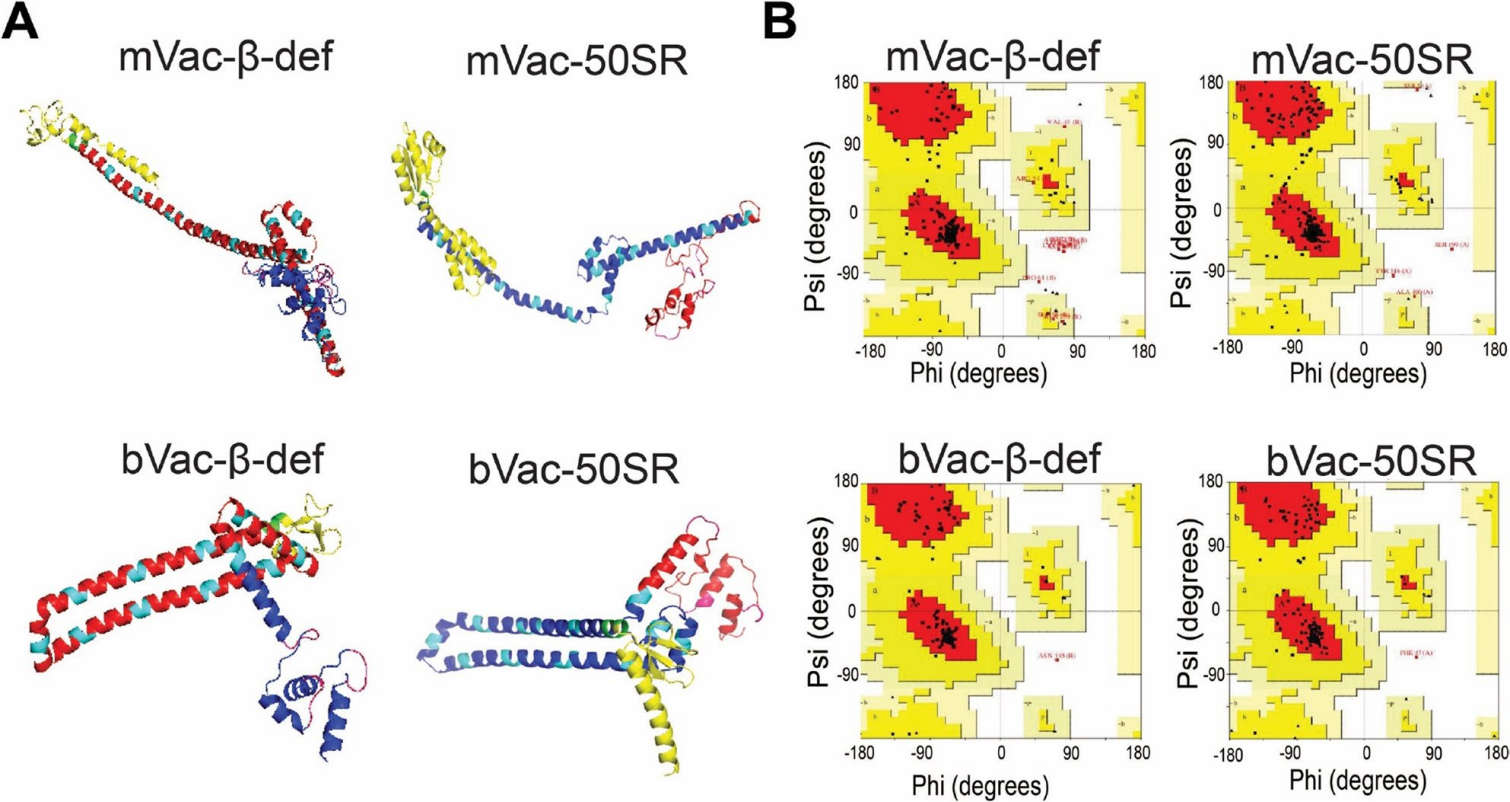
Protein modeling and evaluation of three-dimensional structures of the vaccine constructs. (**A**) The three-dimensional structures of vaccine constructs obtained through protein modeling. (**B**) Ramachandran plots showing the quality of modeled protein structures of vaccine constructs

The vaccine constructs further underwent validation based on their phi (ϕ) and psi (Ψ) torsion angles through Ramachandran plot analysis, which is considered one of the most important approaches in recent vaccine studies [112, 113] **(**Fig. 4A and B**)**. A protein model with over 90% of the residues in the most favorable regions is considered optimal. As depicted in Fig. 4B, the Ramachandran plot analysis for mVac-𝛽-def, mVac-50SR, bVac-𝛽-def, and bVac-50SR-modeled structures reveals values within the favorable regions of 90.7%, 93.3%, 95.9%, and 97%, with additional values falling within the allowed regions of 5.9%, 5.5%, 3.6%, and 2.6%, respectively (Table S5). Moreover, only 1.5%, 0.3%, 0%, and 0% were found in the generously allowed regions, and 1.9%, 0.9%, 0.5%, and 0.4% were scattered in disallowed regions of the Ramachandran plots. These findings confirm the excellent quality of the modeled 3D structures of the vaccine constructs, rendering them highly suitable for molecular docking studies.

We further evaluated the antibody and cytokine profiles elicited by the vaccine candidates through computer-aided immune simulations, as shown in Supplementary Fig. 1. These findings refer to the simulated human immune system due to the unavailability of suitable tools for modeling immune responses in mouse and bovine systems. Despite this limitation, the preliminary computational data suggest that the vaccine candidates designed in this study have the potential to serve as effective immunogens in mouse and bovine hosts. The immune simulation plots indicate high titers of IgM and IgG isotypes, along with robust production of pro-inflammatory cytokines such as IFN-γ and IL-2. This strong cytokine and antibody response suggests the potential activation of B cells and subsequent antibody production, mediated by the T cell-based multi-epitope vaccines. These findings underscore the immunogenic potential of the designed vaccines and their ability to induce both humoral and cellular immune responses.

Additionally, all the vaccine constructs exhibit optimal CAI values of 1.0 and GC content ranging from 49% to 55% (mVac-𝛽-def: 55.0%; mVac-50SR: 52.81%; bVac-𝛽-def: 51.8%; bVac-50SR: 49.53%). These values indicate that the vaccine constructs are highly suitable for efficient recombinant expression in prokaryotic expression systems, particularly in *E. coli*.

### Protein-Protein Docking

**Fig. 5:**
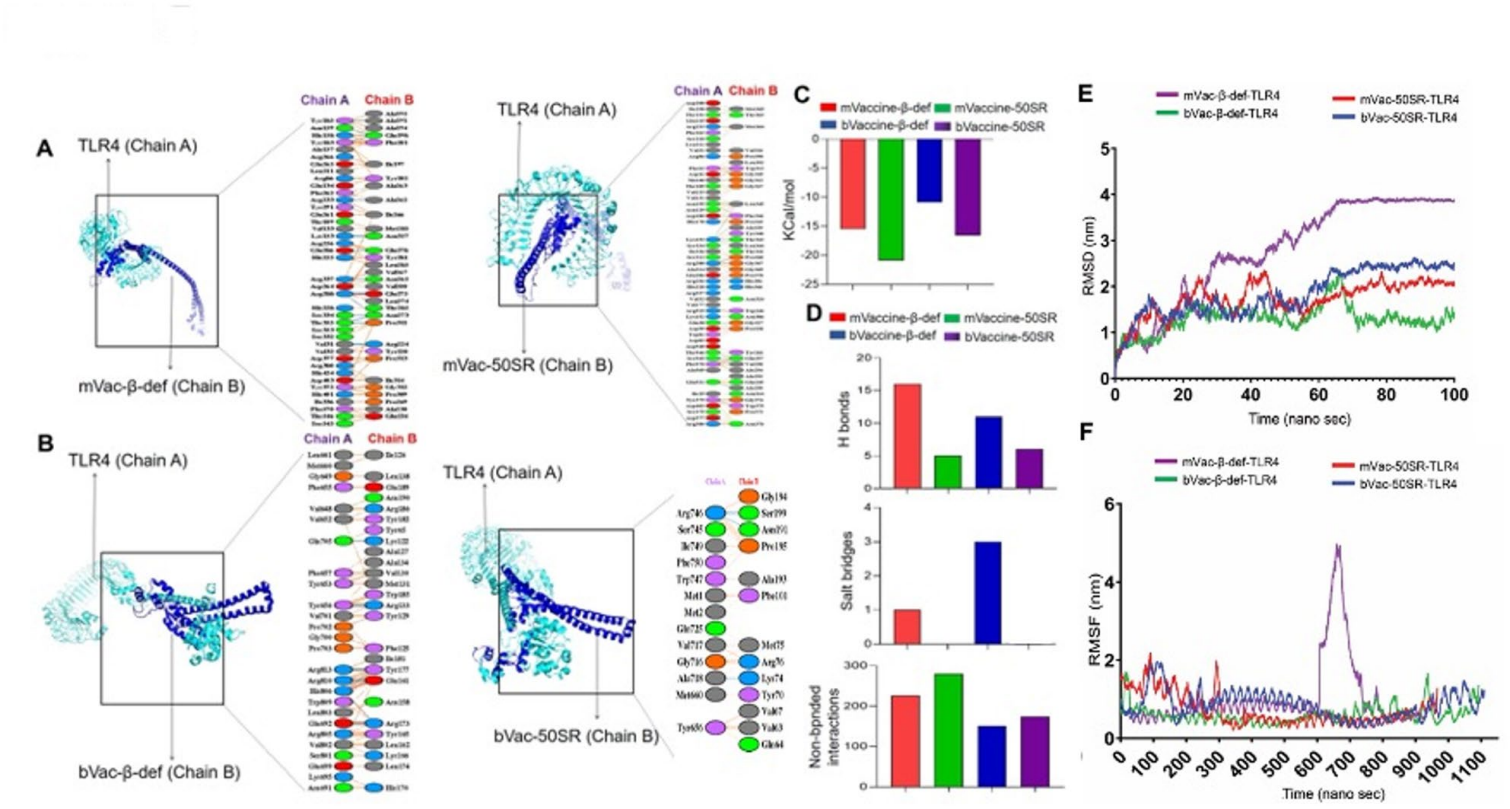
Molecular interactions between the vaccine constructs with mouse and bovine TLR4 structures. **A** Visualization of molecular interactions between the mouse vaccine constructs (chain B) in complex with TLR4 (chain A) are represented in both 3-D and 2-D views. **B** Visualization of molecular interactions between the bovine vaccine constructs (chain B) in complex with TLR4 (chain A) are represented in both 3-D and 2-D views. **C** Bar graph representing the Gibbs free energy (Kcal/mol) values for the murine and bovine vaccine docked complexes. **D** Bar graph representing the total number of hydrogen bonds, salt bridges and non-bonded interactions in the docked complex between the vaccine constructs and TLR4. **E** The graph showing RMSD in the mouse and bovine vaccines and TLR4 docked complexes. **F** The graph representing RMSF of the mouse and bovine vaccine and TLR4 docked complexes

The protein-protein docking is a crucial method for predicting molecular interactions between the TLRs and vaccine constructs [114]. To predict the molecular interaction patterns between all four vaccine constructs (mVac-𝛽-def, mVac-50SR, bVac-𝛽-def, and bVac-50SR) and their receptor, TLR-4, we conducted protein-protein docking using the ClusPro platform. The results revealed binding affinities of −15.5 kcal/mol, −20.9 kcal/mol, −10.9 kcal/mol, and − 16.6 kcal/mol for mVac-𝛽-def, mVac-50SR, bVac-𝛽-def, and bVac-50SR, respectively **(**Fig. 5**)**. Notably, mVac-50SR exhibited the highest negative docking score and confidence score, indicating its superior interaction with the TLR4 molecule.

Further analysis of post-docking interactions revealed a series of molecular interactions between the vaccine constructs and TLR-4, encompassing hydrogen bonds, salt bridges, and non-bonded contacts (Fig. 5A and B). As depicted in Fig. 5A, the docking complex of TLR4 with mVac-𝛽-def vaccine formed 16 hydrogen bonds, 01 salt bridge, and 226 non-bonded interactions. Conversely, the complex of TLR4 and mVac-50SR vaccine exhibited 05 hydrogen bonds and 280 non-bonded interactions. In the case of bVac-𝛽-def vaccine and TLR4, 11 hydrogen bonds, 03 salt bridges, and 151 non-bonded contacts were observed **(**Fig. 5B**)**. Similarly, the docking complex of bVac-50SR vaccine and TLR4 formed 06 hydrogen bonds, and 174 non-bonded contacts **(**Fig. 5B**)**. Notably, this docked complex had the highest number of hydrogen bonds (06), while the murine mVac-50SR and TLR4 complex exhibited the highest number of non-bonded interactions (280).

### Molecular dynamics simulation of docked complexes

MD simulations are one of the well-established methods for obtaining dynamic data at atomic spatial resolution (Gajula et al., 2016). In this study, MD simulations were performed to determine the structural stability of the docking complexes over 100 ns. To analyze the stability of the vaccine constructs in the binding regions of TLRs, root-mean-square deviation (RMSD) and root-mean-square fluctuation (RMSF) plots were calculated using several modules embedded in the Gromacs package. Here, we used representative mVac-50SR and bVac-50SR vaccines to assess their stability. As shown below, overall, the calculated RMSF plots corroborate the findings of the RMSD and docking analyses, indicating that the vaccine constructs interact significantly with their respective TLRs.

RMSD is a widely used method for measuring the stability of simulated systems and is valuable for assessing conformational changes within macromolecular backbone structures during MD simulations on a time scale (Sargsyan et al., 2017). To assess the stability of mVac-50SR and bVac-50SR vaccine constructs binding to their TLR-4, backbone RMSDs were analyzed graphically. As Fig. 5E illustrates, both the docked complexes maintained consistent stability throughout the simulation, with RMSD values ranging from ~ 0.95 to 4.1 nm. The average RMSD values for the mVac-50sR-TLR4 and bVac-50SR-TLR4 docking complexes were 1.72 and 1.83 nm, respectively. These complexes showed small fluctuations between ~ 10 to 60 ns on ~ 0.90 nm, suggesting relatively stable trends with minor conformational changes throughout the 100 ns simulation.

Similarly, to assess the stability of vaccine constructs with β-adjuvants binding to their TLR-4, backbone RMSDs were analyzed graphically. As Fig. 5E illustrates, all the four docked complexes-maintained stability throughout the simulation, with RMSD values ranging from ~ 0.12 to 3.94 nm. The average RMSD values for the mVac-*β*-def-TLR4, mVac-50SR-TLR4, bVac-*β*-def-TLR4 and bVac-50SR-TLR4 docking complexes were 2.77, 1.2, 1.29, 1.83 nm, respectively. These complexes showed initial fluctuations up to ~ 60 ns. After ~ 60 ns, complexes demonstrated stability up to the endpoint without any major fluctuations. As shown in Fig. 5E, the mVac-*β*-def-TLR4 (purple) showed slightly different patterns of fluctuations as compared to the other three complexes. The outcome of the RMSD analysis suggests relatively stable trends with minor conformational changes throughout the 100 ns simulation. Therefore, based on the minimal fluctuations observed and the low differences in computed RMSD scores, it can be inferred that the vaccine-TLR complexes remained stable.

The RMSF plot analysis was conducted to assess the flexibility of individual residues within the simulated systems over time. As a rule, higher fluctuation scores indicate greater flexibility and less stable bonds, while lower values suggest well-structured segments in the protein-protein docking complexes [115]. Figure 5F depicts the calculated RMSF plots for the alpha-carbon atoms of all four simulated systems. As evident from this figure, both complexes, bVac-TLR4 (blue) and mVac-TLR4 (red) demonstrated the close pattern of the peaks between residues 1 to 300 on ~ 2.2 nm. After 300 resides, no significant peak has been observed in these complexes. The average RMSF values for the mVac-50SR-TLR4 and bVac-50SR-TLR4 complexes were 0.73 and 0.85 nm, respectively.

As evident from the Fig. 5F, all four complexes, mVac-*β*-def-TLR4, mVac-50SR-TLR4, bVac-*β*-def-TLR4 and bVac-50SR-TLR4 demonstrated the almost close pattern of the peaks throughout the simulations. However, among these four complexes, the mVac-*β*-def-TLR4 (purple) showed the highest peak between 646 and 675 residues on ~ 4.9 nm. This peak may occur due to the presence of loop in this region. The average RMSF values for the bVac-50SR-TLR4, mVac-50SR-TLR4, bVac-*β*-def-TLR4, and mVac-*β*-def-TLR4 complexes were 0.85, 0.73, 0.60, and 1.03 nm, respectively. All four simulated systems experienced relatively minor conformational changes throughout the simulations.

### Apo simulations of the vaccine constructs on 100 Ns

**Fig. 6:**
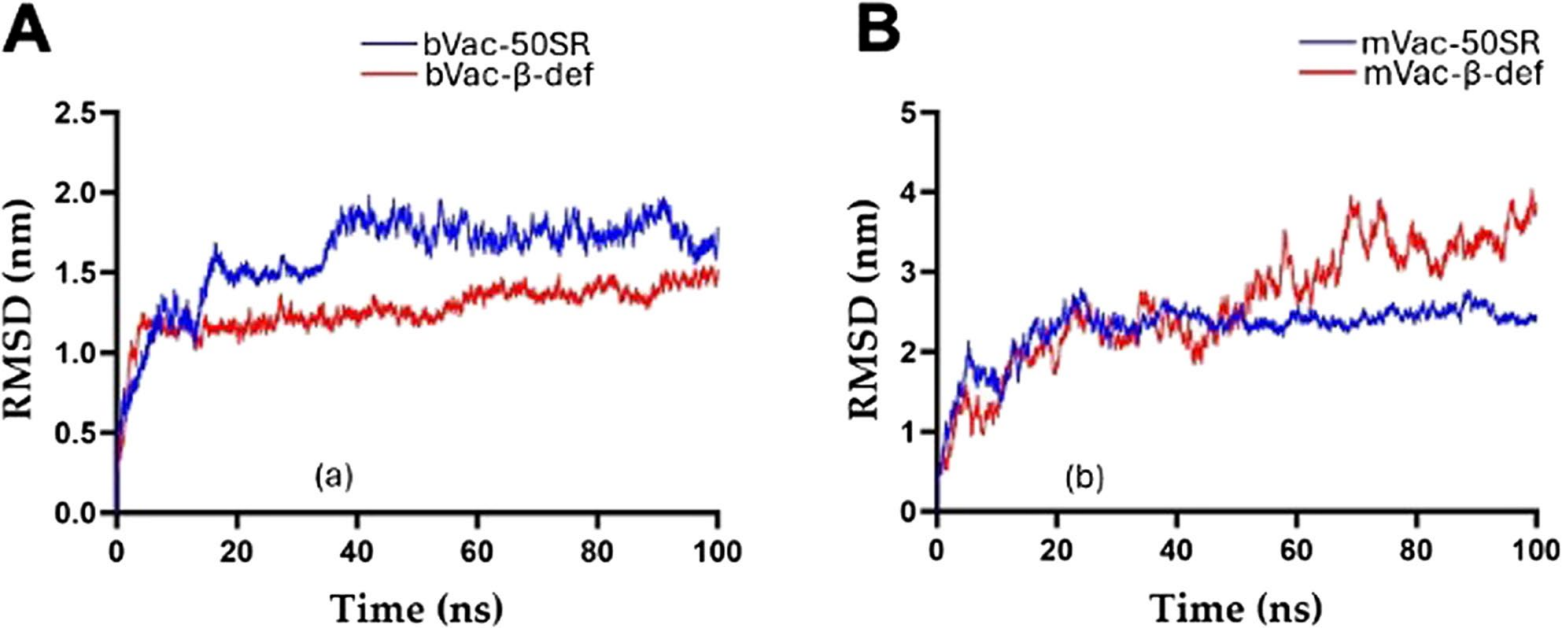
Apo molecular dynamics simulations of (**A**) Bovine vaccine constructs and (**B**) Mouse vaccine constructs

The state-of-the-art apo simulation evaluated the stability and folding conformation of all four bovine and mouse vaccine constructs on 100 ns. The dynamic behavior of the simulated systems was assessed by calculating the root mean square deviation (RMSD). The calculated RMSDs of the designed constructs were graphically measured and discussed. The average RMSD values of the constructs namely bVac-50SR, bVac-β-def, mVac-50-SR, and mVac-β-def were 1.60, 1.26, 2.28, and 2.60 nm, respectively. As depicted in Fig. 6A, the bVac-50SR (blue) showed initial fluctuations up to ~ 40 ns on ~ 1.5 nm. After 40 ns, this construct demonstrated the stability up to 100 ns. On the other hand, no significant fluctuations have been observed in the bVac-β-def (red) case, which showed the stability pattern throughout the simulations on a 100 ns scale. As shown in Fig. 6B, initially both constructs mVac-50-SR and mVac-β-def showed an almost similar pattern of the simulations up to ~ 45 ns, after 45 ns, mVac-β-def (red) demonstrated a fluctuation. This fluctuation didn’t affect the folding conformation of the vaccine construct. Of note, no major fluctuations have been seen in the mVac-50SR (blue) and showed the stability pattern up to the endpoint of the simulations. The outcome of the apo simulations indicated that designed vaccines have minimal conformational changes and reach to a stable folding conformation.

## Discussion

Previous numerous studies on vaccine and immune response against BTV infection highlight the importance of T-cell mediated cross reactive immune response against multiple BTV serotypes due to the presence of conserved T cell epitopes in the BTV antigens [50]. For instance, the viral vectors vaccine candidate carrying the NS1 protein has shown cross reactivity by the CD8 + T cells against various BTV serotypes [116–118]. In addition to this, viral vector vaccines expressing NS2, VP2 and VP7 proteins of BTV also could induce cross protective T-cell mediated immune response against multiple BTV serotypes [119–121]. According to Rojas et al., the conserved epitopes in these proteins could be responsible for both the CD4 + and CD8 + T cell mediated cross-reactive immune response against multiple BTV serotypes [57, 122, 123]. Our approach harnesses this T-cell-mediated cross-reactive phenomenon observed in several immunization strategies and natural BTV infection in development of pan-BTV vaccines.

In pursuit of developing a vaccine that can control all BTV serotypes while minimizing adverse reactions in hosts, our prior research successfully explored and developed pan-BTV vaccines targeting conserved epitopes within BTV nonstructural proteins, specifically NS1 and NS2 [124]. Building upon this foundation, here we sought to extend our efforts to encompass the structural proteins of BTV. Through rigorous epitope selection criteria, we identified highly conserved CD8 + and CD4 + T cell epitopes within VP1, VP5, and VP7 proteins of BTV, and developed in silico-based pan-BTV vaccines capable of conferring protection against all 24 classical serotypes. We excluded VP3 and VP4 proteins from our vaccine design due to their inhibitory nature, which could potentially impede the establishment of robust cross-reactive cellular responses [125].Given the substantial variability among serotypes, the outer capsid of the virus—comprised of VP2 and VP5—is shown to induce serotype-specific neutralizing antibodies [126, 127]. As a result, B cell epitopes have been deliberately omitted from our pan-BTV vaccine design. Instead, we focused on eliciting cell-mediated immune (CMI) responses, as they have demonstrated cross-protection against multiple BTV serotypes in both infections and vaccinations of sheep [5, 20, 127–129]. Notably, viral vectors expressing other BTV proteins such as NS2, VP2, and VP7 have shown to confer CD8 + T cell-mediated cross-protection against various BTV serotypes [119–121]. Moreover, studies by Rojas et al. elegantly demonstrated the relevance of incorporating epitopes from BTV proteins in vaccine formulations [122, 123]. Additionally, the structural proteins of BTV serotypes 25 to 32 were not considered due to incomplete sequence data available on PubMed. Nonetheless, given the inclusion of epitopes present across all 24 serotypes in our vaccine design, it is plausible that they exhibit conservation within the remaining BTV serotypes 25 to 32.

Utilizing computational methods and immunoinformatics not only helps pinpoint essential epitopes but also establishes a strong basis for progressing pan-BTV vaccine development. With meticulous scrutiny, we filtered conserved T cell epitopes based on antigenicity, allergenicity, toxicity, and IFN-𝛾-inducing abilities to pinpoint potential candidates for pan-BTV vaccine design. All four designed vaccine constructs demonstrated an acceptable range of physicochemical properties at the protein sequence level, making them suitable for structural-based evaluation and annotations **(**Table 1**)**. Based on the immunological profiling, all four vaccine constructs have been classified as probable antigens with high antigenic scores and non-allergens or not inducers of autoimmunity in the host. Furthermore, the vaccines elicited strong B cell and cytokine response during immune simulations **(Supplementary Fig. 1)**. This indicates the possibility of B cell activation and antibody production by the T cells via the multi-epitope T cell vaccines. It is important to note that the immune simulation results presented here are based on a human immune system model, as current platforms like C-IMMSIM do not support bovine or ovine immune system modeling. While this represents a limitation in translating the findings directly to the natural hosts of BTV, we believe the simulation still provides useful preliminary insights into the vaccine’s immunogenic potential. Future development of host-specific immune simulation tools—or experimental testing in bovine or ovine systems—will be necessary to better assess the candidate’s applicability in the target species. Based on their Ramachandran plot analysis, all the four vaccine models were found to be stable with high quality and suitable for molecular docking studies. We targeted the TLR4 because of its potential role in skewing anti-viral immune response [96, 130].

The immune system recognizes and responds to antigens through TLRs, with TLR-4 being a prominent pattern recognition receptor that primarily detects LPS from gram-negative bacteria^110^. Beyond LPS, TLR-4 also recognizes protein adjuvants such as the 50 S ribosomal subunit and beta-defensin 2, which are widely utilized in the design and evaluation of multi-epitope vaccines^67–68^. Activation of TLR-4 triggers intracellular signaling cascades, resulting in the production of pro-inflammatory cytokines such as IL-6, IFN- γ, and TNF-α. These cytokines recruit and activate DCs and macrophages at the site of vaccination, promoting antigen uptake, processing, and subsequent presentation to T cells via MHC molecules. This process is critical for the activation of T helper (Th) cells and cytotoxic T lymphocytes (CTLs), which mediate adaptive immunity^111^. The inclusion of TLR-4 agonists in vaccine constructs enhances epitope presentation and overall immune activation, thereby improving vaccine efficacy. In the context of molecular docking, studying TLR-4 interactions provides valuable insights into the binding affinity and interaction patterns of the adjuvant components, which support further refinement of vaccine design and evaluation strategies. The protein-protein docking, and interaction analysis demonstrated that the designed vaccine constructs exhibit a strong molecular interaction pattern with their respective receptor molecules at the structural level. RMSD is a widely used method for measuring the stability of simulated systems and is valuable for assessing conformational changes within macro molecular backbone structures during MD simulations on a time scale [131]. Based on the minimal fluctuations observed and the low differences in computed RMSD scores depicted in the RMSD plots, it can be inferred that the vaccine-TLR complexes remained stable. The RMSF plot analysis was conducted to assess the flexibility of individual residues within the simulated systems over time. As a rule, higher fluctuation scores indicate greater flexibility and less stable bonds, while lower values suggest well-structured segments in the protein-protein docking complexes [115]. Therefore, the calculated RMSF plots corroborate the findings of the RMSD and docking analyses, indicating that the vaccine constructs interact significantly with their respective TLRs.

Overall, through the molecular docking and MD simulation data, we further interpret that the binding affinities observed with the TLR4 fall within the range of reported values for known multi-epitope vaccine candidates, which suggest a favorable vaccine-receptor interaction. Importantly, the docking sites are located adjacent to the TLR4 but not overlapping the dimerization interface, indicating that the vaccine constructs are unlikely to disrupt the receptor function while still enabling immune activation. The MD simulations demonstrate the structural stability of vaccine-TLR4 complexes with low RMSD values supporting their stability under physiological conditions [132].

With a focus on cell-mediated immunity, which has demonstrated its importance in providing cross-protection against various BTV serotypes in prior research [5, 127, 133–140], our vaccine design strategy emphasizes targeting conserved immunogenic cytotoxic T lymphocyte (CTL) and T-helper-1 cell epitopes to develop a highly effective pan-BTV vaccine. Including T-helper epitopes is crucial as CD4 + T-helper-1 cells play a vital role in supporting the function of anti-viral CD8 + CTLs [141–144]. Upon vaccination, antigen-presenting cells (APCs) present epitopes for CD4 + T-helper-1 cell activation, which in turn provide helper signals for CD8 + CTL effector and memory responses [142]. Thus, our vaccine is strategically designed to elicit robust cross-reactive cellular immunity against multiple BTV serotypes.

Our proposed pan-BTV multi-epitope vaccine constructs have several potential advantages over the existing experimental and commercial BTV vaccines, including LAVs, IAVs, VLP-based platforms, and DNA-based vaccines. Unlike LAVs and IAVs, which primarily induce serotype-specific humoral immunity and may pose safety concerns such as reversion to virulence or incomplete inactivation, our constructs are designed to elicit robust T cell–mediated immunity. By incorporating conserved and immunodominant CD4 + and CD8 + T cell epitopes from internal and structural proteins (e.g., VP1, VP5, VP7.), which are more conserved across serotypes than outer capsid proteins, our approach aims to provide broader cross-serotype protection.

Moreover, our constructs exclude allergenic and toxic epitope sequences, enhancing their safety profile over traditional platforms. The use of carefully selected adjuvants, including TLR-4 agonists, supports the activation and maturation of dendritic cells, leading to efficient antigen presentation and subsequent T cell priming [145, 146]. Although antibody responses may still be generated, our design emphasizes cellular immunity as the principal mechanism of protection. Finally, the modular design of our vaccine constructs makes them highly adaptable for delivery via DNA, mRNA, or viral vector platforms, offering translational flexibility that can accommodate different manufacturing capabilities and deployment scenarios. This strategy thus represents a potentially safer, broader, and more versatile alternative to existing BTV vaccine approaches.

One limitation of our study is the inability to develop similar vaccines for sheep, a natural host for BTV, due to the lack of reliable tools for predicting sheep-specific T cell epitopes. Notably, some of the predicted epitopes in our study have been experimentally validated by others [123]. For instance, the MHC class I specific CD8 + T cell epitopes AAGINVGPI, FQGRNDPMM, IQVVFYISM, and BoLA II specific CD4 + T-cell epitope PMMIYLVWRRIENFA in VP7 are reactive against the BTV8 [123].

While the present study provides a robust immunoinformatics framework for the design of a pan-BTV multi-epitope vaccine, we fully acknowledge that all findings remain predictive and require experimental validation. As with all in silico vaccine development efforts, the translational relevance of our approach depends on subsequent in vitro and in vivo investigations. This limitation is important to highlight, as the absence of wet-lab validation currently restricts the immediate applicability of our vaccine constructs.

To move this work forward and strengthen the scientific foundation for preclinical testing, future efforts will focus on the recombinant expression of the selected vaccine candidates in *E. coli* and potentially mammalian expression systems to ensure proper folding and post-translational modifications. Purified proteins will then be assessed in vitro for their ability to activate antigen-presenting cells (APCs), particularly dendritic cells, and to elicit cytokine responses. Importantly, preliminary validation of key predicted epitopes will be pursued through MHC binding assays and T-cell stimulation experiments using either bovine PBMCs or mouse splenocytes, to determine antigen-specific T-cell activation and cytokine profiles.

In vivo testing in small animal models (e.g., mice) and eventually in the natural host (cattle) will further assess immunogenicity, safety, and efficacy, including protection against multiple BTV serotypes. These follow-up studies will be crucial for confirming the immunological potential and practical viability of our constructs. Such a strategy, integrating both predictive and experimental immunology, is aligned with our previously published work on T cell–targeted immunotherapeutics [51], and will ultimately help bridge the gap between computational vaccine design and translational application.

We acknowledge that the lack of species-specific epitope prediction tools for sheep represents a notable limitation, particularly given that sheep are among the most clinically affected hosts of BTV infection. While bovine-based immunoinformatics tools offer a partial proxy, the absence of validated ovine MHC allele databases restricts our ability to accurately predict T cell epitopes and fully assess the cross-species relevance of our vaccine constructs. As emphasized in our previous work [51], addressing this gap through the development of curated sheep-specific MHC class I and II binding prediction algorithms and database resources should be a key priority for future research. Additionally, empirical evaluation of epitope cross-reactivity and immunogenicity in ovine PBMC cultures or sheep challenge models would provide critical data on the vaccine’s efficacy in this primary target species. Incorporating such comparative immunological approaches will be instrumental in translating computational vaccine designs into viable pan-host candidates with real-world applicability in endemic settings.

However, once our in-silico vaccines are experimentally validated, this approach could guide vaccine development for sheep. For instance, a peptide library derived from conserved BTV proteins, such as NS1 and NS2, could be tested using high-throughput ELISpot assays to identify peptides capable of stimulating ovine T cells. These candidate peptides could then be screened for antigenicity, non-allergenicity, and IFN-γ-inducing potential to design a broad-spectrum multi-epitope BTV vaccine for sheep. Additionally, this gap highlights the need for developing user-friendly tools for sheep-specific T cell epitope prediction, which we aim to pursue in future research.

## Conclusion

The challenge of multiple BTV serotypes undermines the effectiveness of current serotype-specific vaccines. Drawing inspiration from broad-spectrum vaccine strategies used in influenza, we have designed in silico pan-BTV multi-epitope vaccines targeting conserved structural proteins to offer coverage across all serotypes. While the lack of reliable tools for predicting T cell epitopes in sheep has limited the development of ovine-specific vaccines, insights from BoLA epitope presentation in cattle help inform our understanding of T cell epitope presentation in sheep. Although our vaccines have only been assessed computationally, making experimental validation essential, this study represents a major advancement in BTV vaccine research. By leveraging cutting-edge in silico tools, we pave the way for the development of vaccines with broad-spectrum protection, improved cross-reactivity, and minimized adverse effects, thus offering a promising foundation for more effective BTV immunoprophylaxis and future research. However, the vaccine constructs require experimental validation before translating them for veterinary applications.

## Supplementary Information

Supplementary Material 1.

## Data Availability

All data generated or analyzed during this study are included in this published article and its supplementary information files. The immunoinformatic prediction outputs (e.g., docked complexes and simulation files) are available from the corresponding author upon reasonable request.

## Abbreviations

TLR: Toll like receptor
RNA: Ribonucleic acid
NS: Nonstructural
LAV: Live attenuated vaccines
IAVs: Inactivated vaccines
DNA: Deoxyribonucleic acid
VLP: Virus-like particle
BoLA: Bovine Leukocyte Antigen
NCBI: National center for Biotechnology information
MEGA: Molecular evolutionary genetic analysis
TAP: Transporter associated antigen processing protein
ERAP: Endoplasmic Reticulum Aminopeptidases
IEDB: AR-Immune epitope database analytical resource
pI: Isoelectric point
II: Instability index
AI: Aliphatic index
GRAVY: Grand average of hydropathicity index
MD: Molecular dynamics
AMBER: Assisted model building with energy refinement
GROMACS: Groningen machine for chemical simulations
LINCS: LINear Constraint Solver
mVac: 𝛽-def-mouse vaccines with either beta-defensin 2 adjuvant
mVac: 50SR-mouse vaccines with 50 S ribosomal subunit adjuvant
bVac: 𝛽-def-bovine vaccines with either beta-defensin 2 adjuvant
bVac: 50SR-bovine vaccines with 50 S ribosomal protein adjuvant
IgM: Immunoglobulin M
IgG: Immunoglobulin G
CAI: Codon adaptation index
GC: Guanine and Cytosine
RMSD: root-mean-square deviation
RMSF: root-mean-square fluctuation
LPS: Lipopolysacharide
IL: 6-Interleukin 6
TNF: α-Tumor necrosis factor alpha
DCs: Dendritic cells
Th: T helper cells
CTLs: cytotoxic T lymphocytes
APCs: antigen-presenting cells
BT: Bluetongue
BTV: Bluetongue virus
CMI: Cell-mediated immunity
MHC: Major histocompatibility complex
VP: Viral protein
IFN: 𝛾-Interferon gamma
