# Supplementary material for: Unleashing the immune arsenal: development of broad spectrum multiepitope bluetongue vaccine targeting conserved T cell epitopes of structural proteins
Source: BMC Genomics. 2026 Apr 17;27:517. doi: 10.1186/s12864-025-12294-2 (PMC13227647; doi:10.1186/s12864-025-12294-2)
Supplement: Supplementary file 1 — Supplementary Material 1. [file 12864_2025_12294_MOESM1_ESM.docx]

Supplementary tables:

**Supplementary Table 1.** MHC class I specific CD8+ T cell epitopes in the BTV1 structural proteins.

| **Allele** | **Peptide** | **ic50** | **Percentile** | **% Conservation** |
| --- | --- | --- | --- | --- |
| **VP1** | | | | |
| H-2-Kb | APYSWFVKM | 286.21 | 0.35 | 100 |
| H-2-Kb | **FQVAYTTPL** | 316.35 | 0.38 | 77.77 |
| H-2-Db |  | 323.75 | 0.11 | 77.77 |
| H-2-Db | IMISKLDEI | 244.42 | 0.1 | 88.88 |
| H-2-Db | LSGENSTLI | 317.09 | 0.11 | 100 |
| H-2-Kb | QSYAFEVYV | 35.61 | 0.07 | 77.77 |
| H-2-Kb | RNYRVSREM | 212.18 | 0.27 | 100 |
| H-2-Kb | RTMMHSALL | 55.52 | 0.09 | 100 |
| H-2-Kb | SFYNESRRM | 455.9 | 0.52 | 88.88 |
| H-2-Kb | SILEFNIKM | 74.56 | 0.13 | 100 |
| H-2-Kb | SNMILASFL | 78.96 | 0.13 | 100 |
| H-2-Kb | SWFVKMWGV | 348.8 | 0.43 | 100 |
| H-2-Kb | TIYSINLSV | 417.48 | 0.49 | 100 |
| H-2-Kb | TKMSFFSKM | 51.13 | 0.09 | 100 |
| H-2-Kb | TSYAKLFDV | 226.02 | 0.29 | 100 |
| H-2-Kb | TTFDAYIRL | 15.62 | 0.03 | 77.77 |
| H-2-Kb | TYLAYPYQL | 457.87 | 0.53 | 88.88 |
| H-2-Kb | VAYTTPLLF | 93.68 | 0.15 | 88.88 |
| H-2-Kb | VIFTKGHTV | 172.27 | 0.24 | 100 |
| H-2-Kb | VISTSYAKL | 63.19 | 0.1 | 88.88 |
| H-2-Kb | VMADFVNRF | 75.21 | 0.13 | 100 |
| H-2-Kb | VMLRAALGL | 204.82 | 0.27 | 66.66 |
| H-2-Kb | VSRGFCHDL | 220.7 | 0.28 | 100 |
| H-2-Kb | VVERFYPGI | 117.0 | 0.18 | 88.88 |
| H-2-Kb | VWCCFINEL | 326.32 | 0.4 | 100 |
| H-2-Kb | YIYKFSDHI | 47.79 | 0.09 | 100 |
| **VP2** | | | | |
| H-2-Kb | FNKWIIAPM | 496.72 | 0.57 | 11.11 |
| H-2-Kb | IAYLEYMVF | 48.23 | 0.09 | 0 |
| H-2-Kb | ILLKFSGHV | 201.43 | 0.27 | 33.33 |
| H-2-Kb | **IMYLNFLPL** | 6.84 | 0.02 | 28.57 |
| H-2-Db |  | 24.69 | 0.03 |  |
| H-2-Kb | INFGRGQKV | 287.47 | 0.35 | 0 |
| H-2-Kb | LNFLPLYFL | 135.11 | 0.2 | 22.22 |
| H-2-Kb | LSIRFQEAI | 228.25 | 0.29 | 33.33 |
| H-2-Db | LSPITADPI | 141.42 | 0.07 | 0 |
| H-2-Kb | MMWNHLVRI | 93.99 | 0.15 | 0 |
| H-2-Kb | RFYDIRPAL | 286.49 | 0.35 | 11.11 |
| H-2-Kb | RTALWYNPI | 323.93 | 0.4 | 0 |
| H-2-Kb | SHRQWSIPL | 494.34 | 0.56 | 22.22 |
| H-2-Kb | TMPEYFNKW | 461.83 | 0.53 | 22.22 |
| H-2-Kb | VIAEFFPTY | 218.09 | 0.28 | 11.11 |
| H-2-Kb | YIYGRVNLF | 245.21 | 0.3 | 33.33 |
| **VP5** | | | | |
| H-2-Kb | EAYREFLNL | 45.54 | 0.08 | 44.44 |
| H-2-Kb | IHFQRRAIL | 90.2 | 0.15 | 66.66 |
| H-2-Kb | VHPIYLGSL | 170.83 | 0.24 | 55.55 |
| H-2-Kb | ISKAFGTQM | 218.11 | 0.28 | 0 |
| H-2-Kb | ILPRFKKAM | 377.89 | 0.46 | 22.22 |
| **VP7** | | | | |
| H-2-Db | AAGINVGPI | 30.04 | 0.03 | 88.88 |
| H-2-Kb | AAIARAAYV | 444.25 | 0.52 | 22.22 |
| H-2-Db | FAMHGVNPM | 41.44 | 0.03 | 33.33 |
| H-2-Db | FQGRNDPMM | 476.48 | 0.13 | 100 |
| H-2-Kb | IAINRYNGL | 13.07 | 0.03 | 100 |
| H-2-Kb | IAWDGQAAL | 320.38 | 0.39 | 66.66 |
| H-2-Kb | IQVVFYISM | 34.24 | 0.07 | 88.88 |
| H-2-Kb | ISPDYTQHM | 83.04 | 0.14 | 88.88 |
| H-2-Kb | KTLNQYPAL | 97.92 | 0.16 | 88.88 |
| H-2-Kb | LTLLLLSTL | 426.67 | 0.5 | 66.66 |
| H-2-Kb | MIYLVWRRI | 130.09 | 0.2 | 77.77 |
| H-2-Kb | RNEMFFMCL | 251.82 | 0.31 | 77.77 |
| H-2-Db | TAIRNRTTL | 7.36 | 0.01 | 77.77 |
| H-2-Kb | TVMRACATL | 233.81 | 0.29 | 66.66 |

**Supplementary Table 2.** MHC class II specific CD4+ T cell epitopes in the BTV1 structural proteins.

| **Allele** | **Peptide Sequence** | **IC50** | **Percentile Rank** | **% Conservation** |
| --- | --- | --- | --- | --- |
| **VP1** | | | | |
| H2-IAb | ERFYPGIAFDVNEGA | 466.10 | 2.30 | 66.66 |
| H2-IAb | RFYPGIAFDVNEGAC | 866.25 | 4.40 | 66.66 |
| H2-IAb | RVVERFYPGIAFDVN | 289.80 | 1.20 | 66.66 |
| H2-IAb | VERFYPGIAFDVNEG | 297.20 | 1.30 | 66.66 |
| H2-IAb | VVERFYPGIAFDVNE | 265.29 | 1.10 | 66.66 |
| H2-IAb | KRVVERFYPGIAFDV | 469.06 | 2.30 | 73.33 |
| H2-IAb | DFQVAYTTPLLFEMC | 856.42 | 4.40 | 86.66 |
| H2-IAb | DVVMRGFITANTILN | 808.13 | 4.10 | 86.66 |
| H2-IAb | EDFQVAYTTPLLFEM | 490.25 | 2.40 | 86.66 |
| H2-IAb | GWNGYGAHPAALNIV | 474.68 | 2.30 | 86.66 |
| H2-IAb | GYGAHPAALNIVMTE | 924.33 | 4.60 | 86.66 |
| H2-IAb | HNFRTGMLQGIREAM | 890.93 | 4.50 | 86.66 |
| H2-IAb | IGWNGYGAHPAALNI | 537.75 | 2.60 | 86.66 |
| H2-IAb | LEDFQVAYTTPLLFE | 490.60 | 2.40 | 86.66 |
| H2-IAb | LGEFSPGRISRTMMH | 661.68 | 3.40 | 86.66 |
| H2-IAb | LPLGEFSPGRISRTM | 886.14 | 4.50 | 86.66 |
| H2-IAb | NGYGAHPAALNIVMT | 700.98 | 3.60 | 86.66 |
| H2-IAb | NLEDFQVAYTTPLLF | 601.58 | 3 | 86.66 |
| H2-IAb | PLGEFSPGRISRTMM | 706.19 | 3.70 | 86.66 |
| H2-IAb | RGFITANTILNVIEK | 598.42 | 3 | 86.66 |
| H2-IAb | SNLEDFQVAYTTPLL | 720.11 | 3.70 | 86.66 |
| H2-IAb | VMRGFITANTILNVI | 677.93 | 3.40 | 86.66 |
| H2-IAb | VVMRGFITANTILNV | 624.48 | 3.20 | 86.66 |
| H2-IAb | WNGYGAHPAALNIVM | 517.40 | 2.50 | 86.66 |
| H2-IAb | INNVLRAPYSWFVKM | 595.08 | 3 | 93.33 |
| H2-IAb | KINNVLRAPYSWFVK | 667.84 | 3.40 | 93.33 |
| H2-IAb | LTRHNFRTGMLQGIR | 742.93 | 3.80 | 93.33 |
| H2-IAb | MRGFITANTILNVIE | 600.99 | 3 | 93.33 |
| H2-IAb | NNVLRAPYSWFVKMW | 624.98 | 3.20 | 93.33 |
| H2-IAb | NVLRAPYSWFVKMWG | 850.26 | 4.30 | 93.33 |
| H2-IAb | PIGWNGYGAHPAALN | 728.61 | 3.80 | 93.33 |
| H2-IAb | RHNFRTGMLQGIREA | 605.93 | 3 | 93.33 |
| H2-IAb | TRHNFRTGMLQGIRE | 714.97 | 3.70 | 93.33 |
| H2-IAb | AGDEFTMSLNVATQD | 950.35 | 4.80 | 100 |
| H2-IAb | DVQGYVRSQVQTMIT | 646.24 | 3.30 | 100 |
| H2-IAb | FFSKMARPAVQAALS | 144.90 | 0.43 | 100 |
| H2-IAb | FSKMARPAVQAALSD | 197.77 | 0.71 | 100 |
| H2-IAb | GDEFTMSLNVATQDF | 816.65 | 4.10 | 100 |
| H2-IAb | KMARPAVQAALSDPQ | 681.37 | 3.50 | 100 |
| H2-IAb | KMSFFSKMARPAVQA | 630.36 | 3.20 | 100 |
| H2-IAb | MSFFSKMARPAVQAA | 189.57 | 0.64 | 100 |
| H2-IAb | QGYVRSQVQTMITKV | 801.94 | 4 | 100 |
| H2-IAb | SFFSKMARPAVQAAL | 134.99 | 0.36 | 100 |
| H2-IAb | SKMARPAVQAALSDP | 400.75 | 1.90 | 100 |
| H2-IAb | TKMSFFSKMARPAVQ | 826.82 | 4.20 | 100 |
| H2-IAb | VQGYVRSQVQTMITK | 541.93 | 2.60 | 100 |
| **VP2** | | | | |
| H2-IAb | EYMVFFPSKAIRLSK | 133.12 | 0.28 | 0 |
| H2-IAb | YMVFFPSKAIRLSKL | 134.60 | 0.29 | 0 |
| H2-IAb | LEYMVFFPSKAIRLS | 164.16 | 0.44 | 0 |
| H2-IAb | YLEYMVFFPSKAIRL | 232.16 | 0.73 | 0 |
| H2-IAb | MVFFPSKAIRLSKLN | 251.06 | 0.86 | 0 |
| H2-IAb | VFFPSKAIRLSKLNE | 479.48 | 2.20 | 0 |
| H2-IAb | QEEYIYGRVNLFDFV | 550.82 | 2.60 | 20 |
| H2-IAb | EQEEYIYGRVNLFDF | 652.44 | 3 | 20 |
| H2-IAb | LDGIVWYLPITHPNK | 733.09 | 3.40 | 13.33 |
| H2-IAb | GIVWYLPITHPNKCI | 771.48 | 3.50 | 13.33 |
| H2-IAb | REQEEYIYGRVNLFD | 819.17 | 3.80 | 20 |
| H2-IAb | FNKWIIAPMFNANVR | 862.69 | 4.10 | 6.66 |
| H2-IAb | EYFNKWIIAPMFNAN | 878.70 | 4.20 | 6.66 |
| H2-IAb | DGIVWYLPITHPNKC | 887.36 | 4.20 | 13.33 |
| H2-IAb | EEYIYGRVNLFDFVA | 888.02 | 4.20 | 20 |
| H2-IAb | NKWIIAPMFNANVRI | 891.32 | 4.20 | 6.66 |
| H2-IAb | YFNKWIIAPMFNANV | 906.44 | 4.30 | 13.33 |
| H2-IAb | GRIRLRFPLSARHLK | 921.80 | 4.30 | 0 |
| H2-IAb | IVWYLPITHPNKCIV | 947.54 | 4.50 | 13.33 |
| H2-IAb | FLDGIVWYLPITHPN | 950.15 | 4.50 | 13.33 |
| **VP5** | | | | |
| H2-IAb | EVPLIGAGMATAVAT | 460.03 | 2.10 | 60 |
| H2-IAb | VPLIGAGMATAVATG | 552.13 | 2.60 | 53.33 |
| H2-IAb | EEVPLIGAGMATAVA | 552.55 | 2.60 | 60 |
| H2-IAb | PLIGAGMATAVATGR | 672.63 | 3.10 | 53.33 |
| H2-IAb | LIGAGMATAVATGRA | 731.67 | 3.40 | 53.33 |
| H2-IAb | MMKFKIPRAQQPQIH | 775.56 | 3.50 | 33.33 |
| H2-IAb | SEEVPLIGAGMATAV | 852.53 | 4 | 53.33 |

| **VP7** | | | | |
| --- | --- | --- | --- | --- |
| H2-IAb | WFMRAAQAATAVVCG | 108.16 | 0.21 | 53.33 |
| H2-IAb | MPWPLTAAIARAAYV | 132.97 | 0.28 | 20 |
| H2-IAb | PMPWPLTAAIARAAY | 170.86 | 0.48 | 26.66 |
| H2-IAb | FMRAAQAATAVVCGP | 189.14 | 0.52 | 53.33 |
| H2-IAb | NPMPWPLTAAIARAA | 270.87 | 0.96 | 26.66 |
| H2-IAb | MDTIAARALTVMRAC | 457.85 | 2.10 | 26.66 |
| H2-IAb | MRAAQAATAVVCGPD | 580.79 | 2.70 | 60 |
| H2-IAb | RIENFAMAQGNSQQT | 671.74 | 3.10 | 53.33 |
| H2-IAb | RPEFAMHGVNPMPWP | 681.15 | 3.10 | 40 |
| H2-IAb | IENFAMAQGNSQQTQ | 689.70 | 3.20 | 46.66 |
| H2-IAb | RRIENFAMAQGNSQQ | 712.81 | 3.30 | 53.33 |

| H2-IAb | LRPEFAMHGVNPMPW | 737.76 | 3.40 | 46.66 |
| --- | --- | --- | --- | --- |
| H2-IAb | DTIAARALTVMRACA | 753.63 | 3.40 | 26.66 |
| H2-IAb | PEFAMHGVNPMPWPL | 842.60 | 3.90 | 40 |
| H2-IAb | VLRPEFAMHGVNPMP | 958.48 | 4.50 | 53.33 |
| H2-IAb | EFAMHGVNPMPWPLT | 987.88 | 4.60 | 40 |
| H2-IAb | WRRIENFAMAQGNSQ | 999.30 | 4.70 | 60 |

**Supplementary Table 3.** BoLA class I specific CD8+ T cell epitopes in the BTV1 structural proteins.

| **Allele** | **Peptide** | **ic50** | **Percentile Rank** | **% Conservation** |
| --- | --- | --- | --- | --- |

| **VP1** | | | | |
| --- | --- | --- | --- | --- |
| BoLA-2:01201 | ASIFDTVAK | 335.84 | 0.03 | 77.77 |
| BoLA-6:01301 | EQYVGDDTL | 459.45 | 0.98 | 100 |
| BoLA-1:02301 | FQSYAFEVY | 361.8 | 0.1 | 100 |
| BoLA-6:01301 | FQVAYTTPL | 33.08 | 0.13 | 77.77 |
| BoLA-1:02301 | FQVAYTTPL | 41.27 | 0.02 | 77.77 |
| BoLA-6:01301 | GLLREFFIL | 174.46 | 0.48 | 88.88 |
| BoLA-1:02301 | GQTFQSYAF | 336.59 | 0.09 | 88.88 |
| BoLA-6:01301 | GSHDRRLPL | 344.1 | 0.8 | 66.66 |
| BoLA-1:02301 | HKKYNSVEL | 298.82 | 0.08 | 88.88 |
| BoLA-6:01301 | ILMLKATFI | 155.29 | 0.44 | 77.77 |
| BoLA-6:01301 | IMISKLDEI | 52.49 | 0.19 | 88.88 |
| BoLA-2:01201 | KATFIGAWK | 407.15 | 0.04 | 77.77 |
| BoLA-6:01301 | KINSRIKAL | 147.88 | 0.43 | 100 |
| BoLA-1:02301 | KKYNSVELY | 203.75 | 0.05 | 100 |
| BoLA-6:01301 | KLGSHDRRL | 372.87 | 0.85 | 66.66 |
| BoLA-6:01301 | KMRMREEDI | 31.07 | 0.12 | 88.88 |
| BoLA-2:01201 | LAYPYQLTK | 295.64 | 0.02 | 88.88 |
| BoLA-1:02301 | LKTVYTTPF | 177.16 | 0.04 | 66.66 |
| BoLA-6:01301 | MLQGIREAM | 435.85 | 0.95 | 100 |
| BoLA-6:01301 | QIMNLVEEL | 344.62 | 0.8 | 88.88 |
| BoLA-6:01301 | QLKPNPSNL | 118.98 | 0.36 | 77.77 |
| BoLA-6:01301 | QMVMLRAAL | 31.71 | 0.12 | 66.66 |
| BoLA-6:01301 | RLQRFRTHV | 99.02 | 0.32 | 88.88 |
| BoLA-6:01301 | RTMMHSALL | 10.04 | 0.04 | 100 |
| BoLA-6:01302 |  | 165.61 | 0.03 | 77.77 |
| BoLA-1:02301 | SLYCTQMVM | 358.7 | 0.1 | 77.77 |
| BoLA-1:02301 | TKMSFFSKM | 117.59 | 0.03 | 100 |
| BoLA-6:01301 | TQGDKRGLI | 368.3 | 0.84 | 100 |
| BoLA-1:02301 | TQMVMLRAA | 493.13 | 0.16 | 66.66 |
| BoLA-6:01301 | VIFTKGHTV | 262.88 | 0.67 | 100 |
| BoLA-6:01301 | VILRKDVVM | 168.79 | 0.47 | 88.88 |
| BoLA-6:01301 | VMLRAALGL | 166.82 | 0.46 | 66.66 |
| BoLA-6:01301 | YIYKFSDHI | 322.84 | 0.77 | 100 |
| BoLA-1:02301 | YKALFRSSF | 81.55 | 0.02 | 88.88 |

| **VP2** | | | | |
| --- | --- | --- | --- | --- |
| BoLA-6:01301 | AQRQSDDPM | 41.47 | 0.15 | 22.22 |
| BoLA-1:02301 |  | 208.36 | 0.05 |  |
| BoLA-4:02401 | CLHTRTMMW | 323.91 | 0.02 | 22.22 |
| BoLA-6:01301 | EQFKMHKIL | 82.9 | 0.27 | 11.11 |
| BoLA-6:01301 | **IMYLNFLPL** | 23.16 | 0.09 | 22.22 |
| BoLA-1:02301 |  | 221.02 | 0.06 |  |
| BoLA-6:01302 |  | 242.34 | 0.04 |  |
| BoLA-1:02301 | KKAGYAEVL | 277.02 | 0.08 | 0 |
| BoLA-6:01301 | KLNEAHAKI | 259.2 | 0.66 | 0 |
| BoLA-6:01301 | KMVEGLTHL | 22.11 | 0.09 | 0 |
| BoLA-6:01301 | KQESIRTAL | 23.48 | 0.1 | 0 |
| BoLA-6:01301 | LLRGYEFTI | 475.52 | 1.0 | 11.11 |
| BoLA-6:01301 | **LQRLTLARF** | 185.19 | 0.5 | 0 |
| BoLA-1:02301 |  | 432.87 | 0.13 |  |
| BoLA-6:01301 | **MMWNHLVRI** | 66.18 | 0.24 | 0 |
| BoLA-1:02301 |  | 477.71 | 0.16 |  |
| BoLA-6:01301 | MVKRTLSPI | 176.98 | 0.48 | 11.11 |
| BoLA-6:01301 | REMRGKEKL | 201.49 | 0.52 | 0 |
| BoLA-6:01301 | RFYDIRPAL | 135.77 | 0.4 | 11.11 |
| BoLA-2:01201 | RGIVQIPKK | 320.87 | 0.03 | 0 |
| BoLA-6:01301 | RGKEKLNVI | 450.51 | 0.97 | 0 |
| BoLA-6:01301 | RLNHSTREI | 363.07 | 0.83 | 0 |
| BoLA-6:01301 | **RQ**W**SI**P**LLL** | 8.3 | 0.03 | 22.22 |
| BoLA-1:02301 |  | 67.84 | 0.02 |  |
| BoLA-6:01302 |  | 97.8 | 0.02 |  |
| BoLA-1:02301 | SHRQWSIPL | 199.5 | 0.05 | 33.33 |
| BoLA-1:02301 | SKKADTMSY | 184.98 | 0.04 | 11.11 |
| BoLA-4:02401 | SMMRSWYDW | 70.78 | 0.01 | 11.11 |
| BoLA-6:01301 | SVRAGRIRL | 447.41 | 0.97 | 0 |
| BoLA-1:02301 | TKLGDVYSM | 476.67 | 0.16 | 33.33 |
| BoLA-6:01301 | TMSYHVEPI | 286.73 | 0.71 | 11.11 |
| BoLA-1:02301 | TRVWWSNPY | 459.52 | 0.15 | 33.33 |
| BoLA-6:01301 | VIRDDIASL | 267.44 | 0.68 | 11.11 |
| BoLA-6:01301 | VMRGKMPEV | 120.88 | 0.36 | 0 |
| BoLA-6:01301 | **VQWMMKDSM** | 52.63 | 0.19 | 0 |
| BoLA-1:02301 |  | 275.16 | 0.08 |  |
| BoLA-2:01201 | VTMPEYFNK | 392.03 | 0.03 | 22.22 |
| BoLA-6:01301 | YIYGRVNLF | 313.75 | 0.76 | 33.33 |
| BoLA-1:02301 | YKRGFPEHL | 448.53 | 0.14 | 0 |
| **VP5** | | | | |

| BoLA-6:01301 | ALKFGCKVL | 494.32 | 1.2 | 44.44 |
| --- | --- | --- | --- | --- |
| BoLA-1:02301 | AQQPQIHVY | 157.2 | 0.04 | 44.44 |
| BoLA-6:01301 | EQRNELVRL | 285.08 | 0.71 | 33.33 |
| BoLA-6:01301 | IMRDRRQMI | 66.47 | 0.24 | 11.11 |
| BoLA-6:01301 | **KLKKVINAL** | 10.3 | 0.04 | 66.66 |
| BoLA-6:01302 |  | 228.41 | 0.04 |  |
| BoLA-6:01301 | **LMHIKNEIL** | 9.57 | 0.03 | 22.22 |
| BoLA-6:01302 |  | 453.76 | 0.06 |  |
| BoLA-4:02401 | QIHVYSAPW | 484.52 | 0.03 | 44.44 |
| BoLA-6:01301 | RAIEGAYKL | 341.11 | 0.8 | 88.88 |
| BoLA-2:01201 | RSLNRFGKK | 173.61 | 0.02 | 66.66 |
| BoLA-1:02301 | SKTVHPIYL | 446.31 | 0.14 | 55.55 |
| BoLA-6:01301 | TQMHTRRLV | 378.46 | 0.85 | 22.22 |
| BoLA-6:01301 | VQGSVHSII | 418.23 | 0.92 | 66.66 |
| **VP7** | | | | |
| BoLA-6:01301 | **AQRNEMFFM** | 55.8 | 0.2 | 66.66 |
| BoLA-1:02301 |  | 142.7 | 0.04 |  |
| BoLA-6:01301 | IAINRYNGL | 446.93 | 0.97 | 100 |
| BoLA-6:01301 | KTLNQYPAL | 155.09 | 0.44 | 88.88 |
| BoLA-6:01301 | SLAQRNEMF | 281.42 | 0.7 | 55.55 |
| BoLA-6:01301 | TLQEARIVL | 382.43 | 0.86 | 77.77 |
| BoLA-6:01301 | TVMRACATL | 264.95 | 0.67 | 66.66 |
| BoLA-6:01301 | VQIQVVFYI | 226.7 | 0.59 | 77.77 |

**Supplementary Table 4.** BoLA class II specific CD4+ T cell epitopes in the BTV1 structural proteins.

| Allele | Peptide | Core_Rel | Score_EL | %Rank_EL | % Conservation |
| --- | --- | --- | --- | --- | --- |
| **VP1** | | | | | |
| BoLA-DRB3_1501 | AALNIVMTEEMYVDS | 0.973 | 0.655263 | 0.94 | 86.66 |
| BoLA-DRB3_1101 | ADPIVVLQSTAGDDR | 0.673 | 0.579204 | 0.84 | 93.33 |
| BoLA-DRB3_1601 | AGDEFTMSLNVATQD | 1.000 | 0.764036 | 0.51 | 100 |
| BoLA-DRB3_0101 | AITVQGAQLIKRVVE | 1.000 | 0.819941 | 0.42 | 100 |
| BoLA-DRB3_1201 | AKLFDVYFEGELDGA | 0.980 | 0.701836 | 0.82 | 80 |
| BoLA-DRB3_1201 | AKNEMQIYGDIPIKV | 1.000 | 0.730536 | 0.72 | 86.66 |
| BoLA-DRB3_2002 | CTQMVMLRAALGLPK | 1.000 | 0.887321 | 0.39 | 73.33 |
| BoLA-DRB3_2002 | DKGFDTLIAATDGSD | 1.000 | 0.869057 | 0.46 | 86.66 |
| BoLA-DRB3_1201 | DVFYDKFLTEPNRYK | 1.000 | 0.822191 | 0.44 | 80 |
| BoLA-DRB3_0101 | EEKILEAVKYSQKLG | 0.660 | 0.656147 | 0.93 | 86.66 |
| BoLA-DRB3_1001 | **ELAEYMTSEKIRFDA** | 1.000 | 0.933189 | 0.07 | 100 |
| BoLA-DRB3_1501 |  | 0.993 | 0.711892 | 0.74 | 100 |
| BoLA-DRB3_1501 | ESILEFNIKMRMREE | 1.000 | 0.639465 | 0.99 | 100 |
| BoLA-DRB3_1601 | EVEWKYVFDGQTFQS | 0.993 | 0.907519 | 0.17 | 80 |
| BoLA-DRB3_1501 | FEKMLKTVYTTPFYP | 1.000 | 0.645331 | 0.97 | 80 |
| BoLA-DRB3_1101 | **FGDMKIDPVGLLREF** | 0.933 | 0.693358 | 0.50 | 73.33 |
| BoLA-DRB3_1601 |  | 1.000 | 0.676099 | 0.76 | 73.33 |
| BoLA-DRB3_2002 | GADPIVVLQSTAGDD | 1.000 | 0.927317 | 0.24 | 93.33 |
| BoLA-DRB3_1501 | GAQLIKRVVERFYPG | 0.980 | 0.728619 | 0.69 | 100 |
| BoLA-DRB3_1201 | HGTKYRRQAEEIIRN | 1.000 | 0.859062 | 0.35 | 73.33 |
| BoLA-DRB3_1501 | HPKKINNVLRAPYSW | 1.000 | 0.811033 | 0.44 | 93.33 |
| BoLA-DRB3_1201 | IREAMAPYRDLRYEG | 1.000 | 0.801094 | 0.49 | 93.33 |
| BoLA-DRB3_1201 | KKKMKIVVTDDAKKR | 0.987 | 0.701870 | 0.82 | 73.33 |
| BoLA-DRB3_0101 | KMKIVVTDDAKKRYK | 0.993 | 0.929056 | 0.13 | 73.33 |
| BoLA-DRB3_1001 | KQGCYVPQDRMMIIS | 1.000 | 0.564207 | 0.74 | 100 |
| BoLA-DRB3_1001 | **KRGLISATKMSFFSK** | 0.993 | 0.677743 | 0.47 | 100 |
| BoLA-DRB3_1501 |  | 0.960 | 0.996306 | 0.00 | 100 |
| BoLA-DRB3_1001 | KTTFDAYIRLDESER | 0.993 | 0.493176 | 0.95 | 73.33 |
| BoLA-DRB3_0101 | LEAVKYSQKLGSHDR | 0.993 | 0.679914 | 0.84 | 73.33 |
| BoLA-DRB3_1601 | LNSWIAQVSMRLGEE | 1.000 | 0.745826 | 0.56 | 86.66 |
| BoLA-DRB3_1601 | LSEQYVGDDTLFYAK | 1.000 | 0.645652 | 0.86 | 93.33 |
| BoLA-DRB3_1001 | PNRYKALFRSSFYNE | 1.000 | 0.788183 | 0.27 | 80 |
| BoLA-DRB3_1101 | QAEEIIRNISLRKER | 0.933 | 0.590924 | 0.81 | 73.33 |
| BoLA-DRB3_0101 | QMVMLRAALGLPKKK | 0.973 | 0.662581 | 0.91 | 73.33 |
| BoLA-DRB3_1201 | QPGILTFLSEQYVGD | 0.987 | 0.892557 | 0.27 | 86.66 |
| BoLA-DRB3_1001 | **RDLRYEGYTLEQIID** | 1.000 | 0.577280 | 0.70 | 93.33 |
| BoLA-DRB3_1201 |  | 1.000 | 0.921555 | 0.21 | 93.33 |
| BoLA-DRB3_0101 | REFFKQAYVEAKERR | 0.993 | 0.737498 | 0.66 | 100 |
| BoLA-DRB3_1101 | RKERLYGIPVLDEVE | 1.000 | 0.841413 | 0.18 | 73.33 |
| BoLA-DRB3_1601 | RRLFKTTFDAYIRLD | 0.993 | 0.683598 | 0.74 | 80 |
| BoLA-DRB3_1001 | SRIKALVIFTKGHTV | 1.000 | 0.709426 | 0.41 | 100 |
| BoLA-DRB3_1201 | SYAFEVYVNSILPWS | 1.000 | 0.907209 | 0.24 | 93.33 |
| BoLA-DRB3_1201 | TKGHTVFTDEELHKK | 1.000 | 0.902600 | 0.25 | 93.33 |
| BoLA-DRB3_0101 | TPYSVEKTQTHAKQG | 1.000 | 0.889602 | 0.23 | 100 |
| BoLA-DRB3_1501 | VDSIMISKLDEIMAP | 0.993 | 0.807055 | 0.45 | 93.33 |
| BoLA-DRB3_1501 | VEKFIEFRAKNEMQI | 1.000 | 0.704441 | 0.77 | 86.66 |
| BoLA-DRB3_1101 | VPKIKVLKKLIDPNR | 0.993 | 0.537653 | 0.84 | 100 |
| BoLA-DRB3_1501 | VSSVDVANRIAVDKG | 1.000 | 0.954346 | 0.11 | 73.33 |

| **VP2** | | | | | |
| --- | --- | --- | --- | --- | --- |
| BoLA-DRB3_1201 | DHELEIFGESIVDIS | 1.000 | 0.684825 | 0.88 | 0 |
| BoLA-DRB3_1201 | DVAYGQMINEMINGG | 1.000 | 0.889147 | 0.28 | 6.66 |
| BoLA-DRB3_0101 | KPTYDIVVHAERRDR | 0.620 | 0.649564 | 0.96 | 6.66 |
| BoLA-DRB3_1001 | MPEYFNKWIIAPMFN | 1.000 | 0.733469 | 0.37 | 20 |
| BoLA-DRB3_1101 | NKCIVAIEVSDERVP | 1.000 | 0.971167 | 0.02 | 6.66 |
| BoLA-DRB3_1601 | PLYFLVGDNMIYSHR | 0.567 | 0.791218 | 0.45 | 20 |
| BoLA-DRB3_1201 | REMLKYYANTTVYDG | 0.993 | 0.852492 | 0.37 | 6.66 |
| BoLA-DRB3_1601 | SIRFQEAIDNKFRQH | 1.000 | 0.846016 | 0.31 | 26.66 |
| BoLA-DRB3_1201 | TMSYHVEPIEDASKG | 1.000 | 0.809814 | 0.47 | 6.66 |
| BoLA-DRB3_1601 | VLTIDFEKDAKLTTN | 0.993 | 0.946640 | 0.09 | 13.33 |
| BoLA-DRB3_0101 | YDIVVHAERRDRSQP | 0.993 | 0.868815 | 0.28 | 0 |
| BoLA-DRB3_0101 | YSEGIVSHRVCKKNL | 1.000 | 0.892155 | 0.23 | 0 |
| **VP5** | | | | | |
| BoLA-DRB3_2002 | AYREFLNLAISKAFG | 1.000 | 0.889590 | 0.38 | 20 |
| BoLA-DRB3_0101 | GKVIRSLNRFGKKVG | 0.973 | 0.652679 | 0.94 | 60 |
| BoLA-DRB3_0101 | GRAIEGAYKLKKVIN | 1.000 | 0.991217 | 0.01 | 73.33 |
| BoLA-DRB3_1201 | IKEKFEKELEEVYNF | 1.000 | 0.671822 | 0.93 | 26.66 |
| BoLA-DRB3_1601 | KNAIEVERDGMQEEA | 1.000 | 0.799382 | 0.43 | 26.66 |
| BoLA-DRB3_1201 | LEEVYNFYNGEANAE | 0.880 | 0.679128 | 0.90 | 20 |
| BoLA-DRB3_2002 | LKKVINALSGIDLTH | 1.000 | 0.823960 | 0.65 | 66.66 |
| BoLA-DRB3_1001 | LTEAYREFLNLAISK | 1.000 | 0.778537 | 0.29 | 26.66 |
| BoLA-DRB3_1201 | NHKELMHIKNEILPR | 1.000 | 0.868211 | 0.33 | 33.33 |
| BoLA-DRB3_1001 | NKAVTSYNKILTEED | 1.000 | 0.766740 | 0.31 | 26.66 |

| BoLA-DRB3_1101 | PDNALAVSVLIKERA | 1.000 | 0.716061 | 0.44 | 20 |
| --- | --- | --- | --- | --- | --- |
| BoLA-DRB3_1101 | RDKIDALKNAIEVER | 0.993 | 0.566900 | 0.88 | 46.66 |
| BoLA-DRB3_0101 | RPSVVSTILEYRAKE | 0.987 | 0.720759 | 0.71 | 13.33 |
| BoLA-DRB3_1201 | SIDLVHYEDLTAHAH | 0.993 | 0.854740 | 0.36 | 40 |
| BoLA-DRB3_1201 | TEAYREFLNLAISKA | 1.000 | 0.970626 | 0.10 | 26.66 |
| BoLA-DRB3_1001 | VRLKYNDKIKEKFEK | 0.820 | 0.516307 | 0.88 | 20 |
| BoLA-DRB3_1601 | YNFYNGEANAEIEDE | 1.000 | 0.822089 | 0.37 | 13.33 |
| **VP7** | | | | | |
| BoLA-DRB3_2002 | IAINRYNGLTLRGVT | 1.000 | 0.836355 | 0.60 | 100 |
| BoLA-DRB3_1501 | **LADVYTVLRPEFAMH** | 1.000 | 0.731772 | 0.68 | 66.66 |
| BoLA-DRB3_2002 |  | 1.000 | 0.864128 | 0.48 |  |
| BoLA-DRB3_1001 | PMMIYLVWRRIENFA | 0.993 | 0.649253 | 0.53 | 73.33 |
| BoLA-DRB3_1601 | QVVFYISMDKTLNQY | 1.000 | 0.911634 | 0.16 | 93.33 |
| BoLA-DRB3_0101 | RGVTMRPTSLAQRNE | 1.000 | 0.709026 | 0.75 | 66.66 |
| BoLA-DRB3_1201 | RPEFAMHGVNPMPWP | 1.000 | 0.754583 | 0.64 | 46.66 |
| BoLA-DRB3_1501 | TIGVLATPEIPFTTE | 1.000 | 0.635758 | 1.00 | 93.33 |

**Supplementary Table 5.** List of finalized CD8+ and CD4+ T cell epitopes for the design of pan-BTV multi-epitope mouse vaccine.

| **Mouse CD8+ T cell epitopes** | **Allele** | **Epitope** | **Antigenicty**  **score** | **Antigenicty** | **Allergenicity** | **Toxicity** |  |
| --- | --- | --- | --- | --- | --- | --- | --- |
|  | **VP1** | | | | | |  |
|  | **H-2-Kb** | TKMSFFSKM | **1.1372** | Antigen | Non-allergen | Non-Toxin |  |
|  | **H-2-Kb** | VISTSYAKL | **0.5864** | Antigen | Non-allergen | Non-Toxin |  |
|  | **H-2-Kb** | SILEFNIKM | **1.6253** | Antigen | Non-allergen | Non-Toxin |  |
|  | **H-2-Kb** | VAYTTPLLF | **0.7449** | Antigen | Non-allergen | Non-Toxin |  |
|  | **H-2-Kb** | VMLRAALGL | **0.6255** | Antigen | Non-allergen | Non-Toxin |  |
|  | **H-2-Db** | IMISKLDEI | **0.5623** | Antigen | Non-allergen | Non-Toxin |  |
|  | **VP5** | | | | | |  |
|  | **H-2-Kb** | IHFQRRAIL | 1.5373 | Antigen | Non-allergen | Non-Toxin |  |
|  | **VP7** | | | | | |  |
|  | **H-2-Db** | AAGINVGPI | 1.5614 | Antigen | Non-allergen | Non-Toxin |  |
|  | **H-2-Db** | FQGRNDPMM | 1.2268 | Antigen | Non-allergen | Non-Toxin |  |
|  | **H-2-Kb** | IAWDGQAAL | 0.7831 | Antigen | Non-allergen | Non-Toxin |  |
|  | **H-2-Kb** | IQVVFYISM | 0.8668 | Antigen | Non-allergen | Non-Toxin |  |
|  | **H-2-Kb** | LTLLLLSTL | 0.6520 | Antigen | Non-allergen | Non-Toxin |  |
|  | **H-2-Db** | TAIRNRTTL | 0.8312 | Antigen | Non-allergen | Non-Toxin |  |
| **Mouse CD4+ T cell epitopes** | **VP1** | | | | | |  |
|  | **Allele** | **Epitope** | **Antigenicity**  **score** | **Antigenicity** | **Allergenicity** | **Toxicity** | **IFNg inducing** |
|  | **H2-IAb** | GWNGYGAHPAALNIV | 0.7697 | Antigen | Non-allergen | Non-Toxin | Positive |
|  | **H2-IAb** | NLEDFQVAYTTPLLF | 0.9151 | Antigen | Non-allergen | Non-Toxin | Positive |
|  | **H2-IAb** | LGEFSPGRISRTMMH | 0.7394 | Antigen | Non-allergen | Non-Toxin | Positive |
|  | **H2-IAb** | PIGWNGYGAHPAALN | 0.8943 | Antigen | Non-allergen | Non-Toxin | Positive |
|  | **VP5** | | | | | |  |
|  | **H2-IAb** | EEVPLIGAGMATAVA | 0.5161 | Antigen | Non-allergen | Non-Toxin | Positive |

**Supplementary Table 6.** List of finalized CD8+ and CD4+ T cell epitopes for the design of pan-BTV multi-epitope bovine vaccine.

| **Bovine CD8+ T**  **cell epitopes** | **Allele** | **Epitope** | **Antigenicity score** | **Antigenicty** | **Allergenicity** | **Toxicity** | |
| --- | --- | --- | --- | --- | --- | --- | --- |
|  | **VP1** | | | | | | |
|  | BoLA-6:01301 | KMRMREEDI | 1.7662 | Antigen | Non-allergen | Non-Toxin | |
|  | BoLA-6:01301 | IMISKLDEI | 0.5623 | Antigen | Non-allergen | Non-Toxin | |
|  | BoLA-1:02301 | TKMSFFSKM | 1.1372 | Antigen | Non-allergen | Non-Toxin | |
|  | BoLA-6:01301 | KINSRIKAL | 1.1637 | Antigen | Non-allergen | Non-Toxin | |
|  | BoLA-6:01301 | ILMLKATFI | 1.1657 | Antigen | Non-allergen | Non-Toxin | |
|  | BoLA-6:01301 | VMLRAALGL | 0.6255 | Antigen | Non-allergen | Non-Toxin | |
|  | BoLA-1:02301 | KKYNSVELY | 0.5682 | Antigen | Non-allergen | Non-Toxin | |
|  | BoLA-1:02301 | HKKYNSVEL | 1.1492 | Antigen | Non-allergen | Non-Toxin | |
|  | BoLA-6:01301 | KLGSHDRRL | 1.5803 | Antigen | Non-allergen | Non-Toxin | |
|  | **VP7** | | | | | | |
|  | BoLA-6:01301 | TLQEARIVL | 0.5255 | Antigen | Non-allergen | Non-Toxin | |
|  | BoLA-6:01301 | VQIQVVFYI | 1.0097 | Antigen | Non-allergen | Non-Toxin | |
|  | **Allele** | **Epitope** | **Antigenicity score** | **Antigenicit**  **y** | **Allergenicity** | **Toxicit**  **y** | **IFNg**  **inducing** |
|  | **VP1** | | | | | | |
|  | BoLA-DRB3_0101 | QMVMLRAALGLPK KK | 0.7323 | Antigen | Non-allergen | Non-  Toxin | Positive |
|  | BoLA-DRB3_1101 | ADPIVVLQSTAGDD R | 0.5582 | Antigen | Non-allergen | Non-  Toxin | Positive |
|  | **VP5** | | | | | | |

| **Bovine CD4+ T**  **cell epitopes** | BoLA-DRB3_1201 | SIDLVHYEDLTAHA H | 1.0504 | Antigen | Non-allergen | Non- toxin | Positive |
| --- | --- | --- | --- | --- | --- | --- | --- |
|  | **VP7** | | | | | | |
|  | BoLA-DRB3_1001 | PMMIYLVWRRIENF A | 0.7744 | Antigen | Non-allergen | Non- Toxin | Positive |

**Supplementary Table 7.** Ramachandran plot analysis of the designed vaccine constructs.

|  | **mVac-β-def** | **mVac-50s R** | **bVac-β-def** | **bVac-50s R** |
| --- | --- | --- | --- | --- |
| **Most favoured** | 90.7 % | 93.3 % | 95.9 % | 97 % |
| **Additionally allowed** | 5.9 % | 5.5 % | 3.6 % | 2.6 % |
| **Generally allowed** | 1.5 % | 0.3 % | 0.0 | 0.0 % |
| **Disallowed** | 1.9 % | 0.9 % | 0.5 % | 0.4 % |
